# Gene co-expression network analysis identifies porcine genes associated with variation in metabolizing fenbendazole and flunixin meglumine in the liver

**DOI:** 10.1038/s41598-017-01526-5

**Published:** 2017-05-02

**Authors:** Jeremy T. Howard, Melissa S. Ashwell, Ronald E. Baynes, James D. Brooks, James L. Yeatts, Christian Maltecca

**Affiliations:** 1 Department of Animal Science, North Carolina State University, Raleigh, NC 27695-7621 USA; 2 Department of Population Health and Pathobiology, Center for Chemical Toxicology and Research Pharmacokinetics, North Carolina State University, College of Veterinary Medicine, 4700 Hillsborough Road, Raleigh, North Carolina 27606 USA

## Abstract

Identifying individual genetic variation in drug metabolism pathways is of importance not only in livestock, but also in humans in order to provide the ultimate goal of giving the right drug at the right dose at the right time. Our objective was to identify individual genes and gene networks involved in metabolizing fenbendazole (FBZ) and flunixin meglumine (FLU) in swine liver. The population consisted of female and castrated male pigs that were sired by boars represented by 4 breeds. Progeny were randomly placed into groups: no drug (UNT), FLU or FBZ administered. Liver transcriptome profiles from 60 animals with extreme (i.e. fast or slow drug metabolism) pharmacokinetic (PK) profiles were generated from RNA sequencing. Multiple cytochrome P450 (*CYP1A1*, *CYP2A19 and CYP2C36)* genes displayed different transcript levels across treated versus UNT. Weighted gene co-expression network analysis identified 5 and 3 modules of genes correlated with PK parameters and a portion of these were enriched for biological processes relevant to drug metabolism for FBZ and FLU, respectively. Genes within identified modules were shown to have a higher transcript level relationship (i.e. connectivity) in treated versus UNT animals. Investigation into the identified genes would allow for greater insight into FBZ and FLU metabolism.

## Introduction

Drug use and how it is currently regulated in livestock has received increased attention due to animal welfare concerns, food safety and the prevalence of antibiotic resistance^1^. Furthermore, minimum withdrawal times are based on pharmacokinetic (PK) studies involving a small number of healthy animals and generalized to the entire population, which disregards factors that may alter drug metabolism such as disease status, breed or sex of the treated animal^2–4^. As a result, drug residue violation in food animals has become a global food safety concern^4, 5^. Previous work has been conducted on variation in gene expression across pigs for multiple cytochrome P450 genes with functions related to drug metabolism^6, 7^, although gene expression levels across the whole transcriptome following drug administration has not been reported. The identification of genes or gene networks that display altered expression levels following drug administration and impact the metabolism of the drug not only provides insights into livestock drug metabolism, but also human drug metabolism. The commercial pig as an animal model in biomedical research has become increasingly relevant, due to the fact that the anatomy, genetics and physiology reflect human biology more closely than classic animal models such as fruit fly, zebrafish and rodents^8^. Furthermore, the drug classes (i.e. common molecular mechanism of action) utilized in human medicine are also used to treat livestock therefore information generated from swine research could impact both livestock and human drug development and allow for more effective drug administration^1^. Therefore using the commercial pig as a model to gain insight into drug-metabolizing enzyme biology would allow for both livestock and human medicine to move closer to the ultimate goal of giving the right drug at the right dose at the right time.

The drugs fenbendazole (FBZ) and flunixin meglumine (FLU) are utilized across a variety of livestock species, although they are not cleared for use in humans^2^. The broad-spectrum drug FBZ is utilized as an antihelmitic drug and is often administered as a feed additive. It undergoes CYP450-mediated oxidation and conjugation with glucoronide and sulfate^9^. Flunixin meglumine is a drug used for the control of pyrexia associated with swine respiratory disease and works by decreasing prostaglandin synthesis by inhibiting the cyclo-oxygenase enzyme^10^. The objective of the current study was to integrate gene co-expression networks, gene versus drug metabolizing parameter correlations and differential transcript analysis for animals administered FBZ or FLU to identify genes and gene networks that impact drug metabolism.

## Results

### The genetic background of selected animals

**Figure 1:**
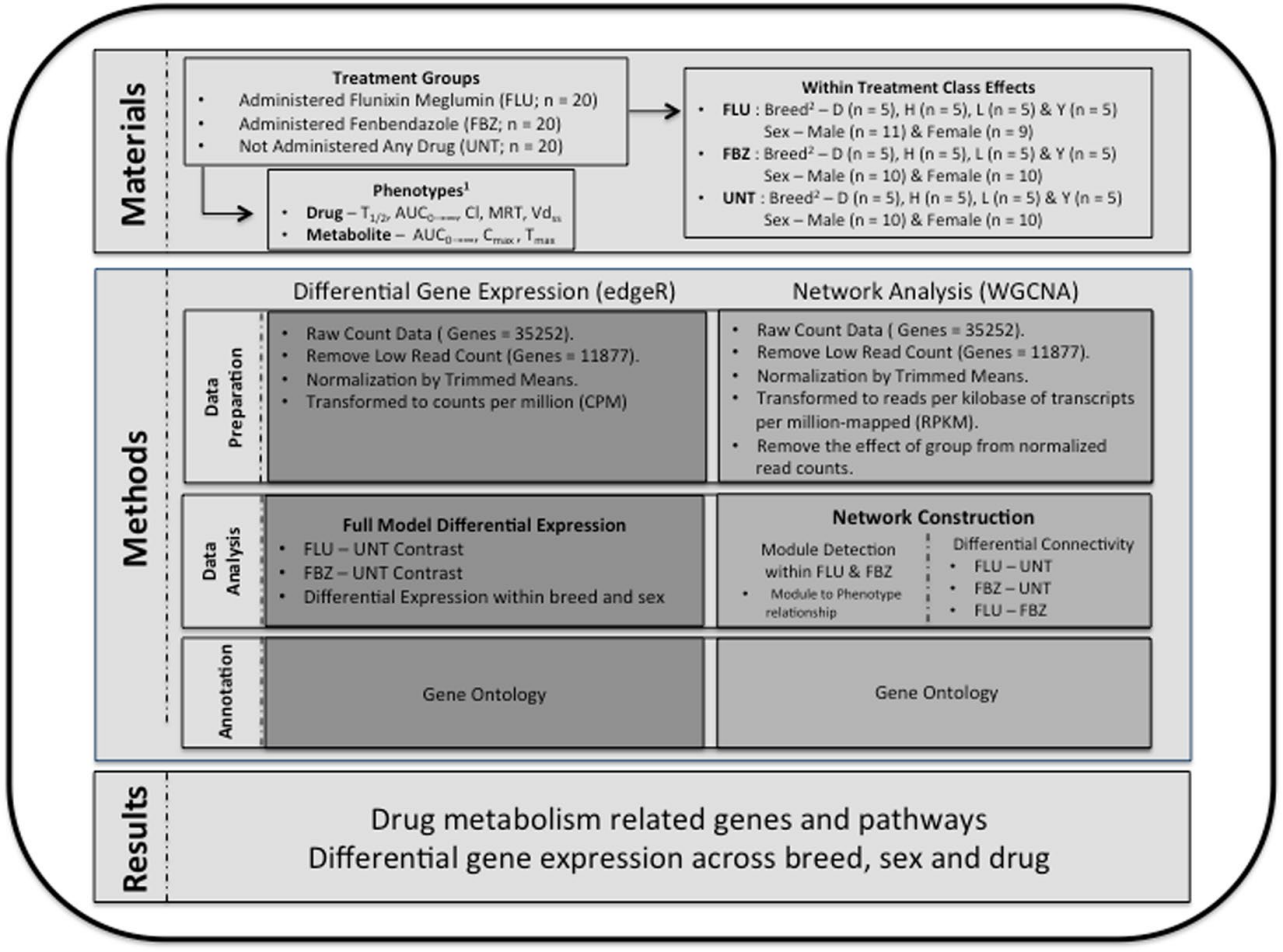
Number of animals within each treatment and workflow of RNA-Seq data analysis. ^1^The pharmacokinetic (PK) phenotypes were half-life (T_1/2_; h), area under the plasma concentration-time curve from time zero to infinity (AUC_0→∞_; h*μg/mL), clearance (Cl; L/h/kg), mean residence time (MRT; h), volume of distribution at steady state (Vd_ss;_ L/kg), peak concentration (C_max_; *μ*g/mL) and time at which maximum concentration occurs (T_max_; h). ^2^Refers to breed and D = Duroc; H = Hampshire; L = Landrace; Y = Yorkshire.

The pigs utilized in the current study were derived from a large resource population (n = 229) that were administered FLU or FBZ^2, 7^. The animals in the full resource population were spread across 5 batches. Across all animals an initial dose of FLU or FBZ was administered to estimate PK parameters. A second dose was administered and one hour after drug administration, animals were sacrificed and a liver sample was collected. The pigs chosen for the current study displayed extreme (i.e. fast or slow drug metabolism) drug clearance PK parameters for FLU (n = 20) and FBZ (n = 20). Furthermore, liver samples were collected from animals that did not receive any drug administered (UNT; n = 20). The pigs were crossbred females or castrated males and the breeds of the sires were Duroc (D), Hampshire (H), Landrace (L) or Yorkshire (Y). The number of animals within each drug, breed and sex class is outlined at the top of Fig. 1 under the Materials section. The range in clearance (L/h/kg) PK parameter values was 0.035 to 1.818 (mean = 0.27 ± 0.22) and 0.046 to 0.271 (mean = 0.12 ± 0.04) for FLU and FBZ, respectively. A low clearance value indicates the drug remains in the blood plasma longer as compared to an animal that has a higher clearance value. The variation across multiple PK parameters, including clearance, is displayed in Figures S1 and S2 for FBZ and FLU, respectively. For the pigs selected in the current study, liver transcriptome profiles were generated from RNA sequencing (RNA-Seq). The degree of similarity in the transcriptome between animals administered either FBZ or FLU versus UNT based on to top 500 genes with the largest transcript standard deviation is outlined in Figure S3. As illustrated in Figure S3 there is evidence across both drugs that transcript levels were altered, based on 500 genes with the largest transcript standard deviation, for animals treated versus animals untreated.

**Figure 2:**
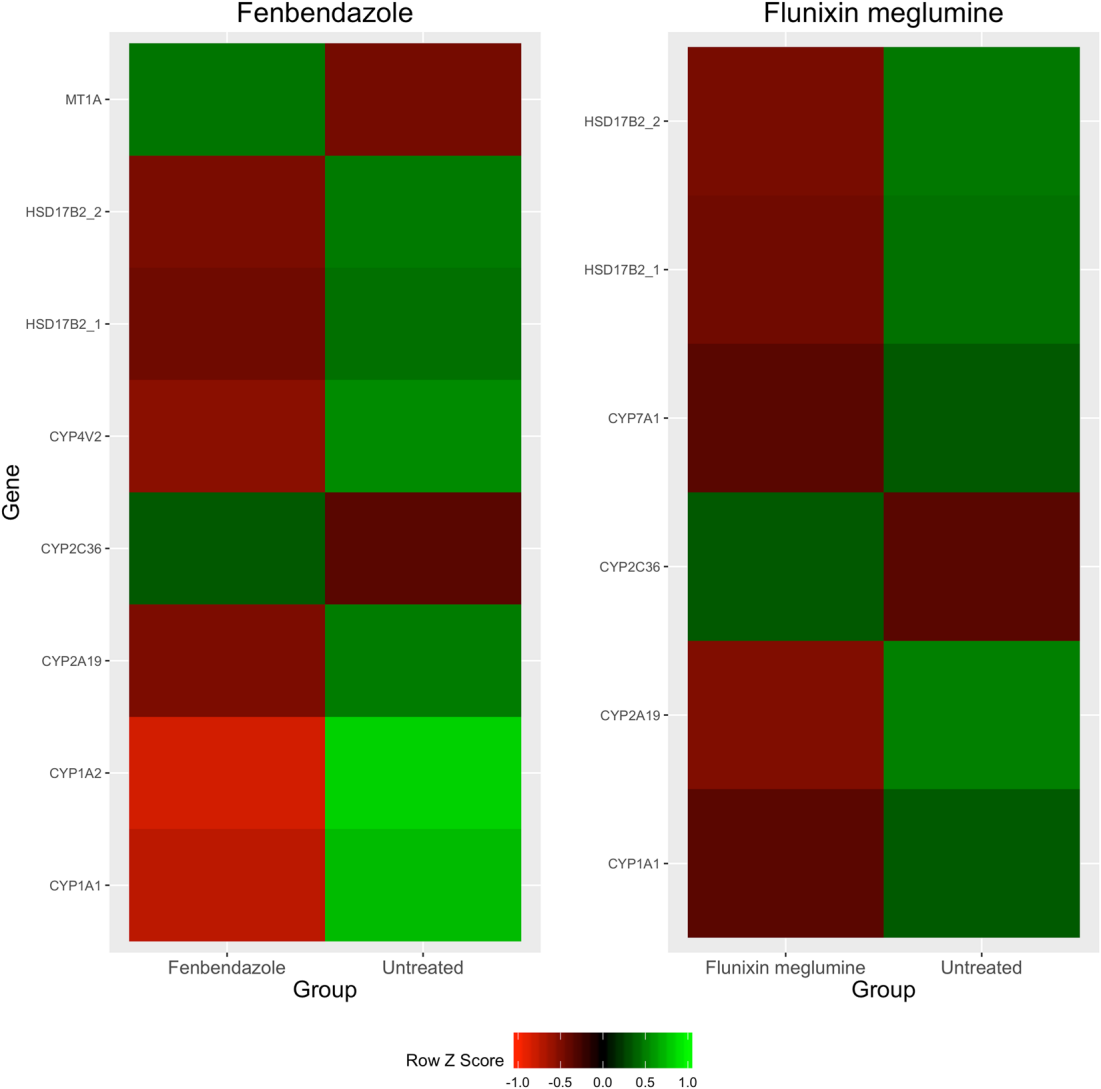
Expression profiles of genes with known Phase I and II drug metabolism functions and highly significant (FDR < 0.0001) transcript differences across animals administered a drug versus untreated.

### Transcript level differences between treated and untreated animals

Differential transcript analysis was conducted between animals administered FBZ or FLU versus UNT animals using the edgeR package^11^ (version 3.8.6). Genes with low transcript counts were removed from the analysis, which resulted in 11,877 genes remaining for analysis. The count data generated from RNA-Seq were normalized and transformed to counts per million (CPM). An overall contrast (i.e. across breed and sex) between FBZ or FLU versus UNT based on normalized CPM values were conducted along with pairwise contrasts within sex and breed. The effect of batch was accounted for in the breed and sex contrasts. Genes were investigated if they had false discovery rate (FDR) value less than 0.05.

The total number of genes that had significant transcript differences (FDR < 0.05) across animals administered a drug versus untreated was 819 and 1153 for FBZ and FLU, respectively. Annotated genes of high significance (FDR < 0.0001) within each drug were investigated and multiple CYP450 genes with functions related to Phase I drug metabolism were identified for FBZ (*CYP1A1*, *CYP2A19*, *CYP2C36* and *CYP4V2*) and FLU (*CYP1A1*, *CYP2A19*, *CYP2C36* and *CYP7A1*). Furthermore, genes involved in Phase II drug metabolism were identified for FBZ (*HSD17B2* and *MT1A*) and FLU (*HSD17B2*). Expression profiles of significant transcript level differences across treated versus control involved in Phase I or II drug metabolism are outlined in Fig. 2. Of the genes found to be highly significant in the across breed and sex contrast for FBZ, the majority of the CYP450 genes were found to display significant (FDR < 0.0001) pairwise breed contrasts including *CYP1A1*, *CYP2A19* and *CYP2C36*. Furthermore, an additional CYP450 gene, *CYP2B22*, that was not significant in the across breed contrast, was significant in the pairwise contrast for FBZ. For FLU, *CYP2C36* displayed significant pairwise breed contrasts and two additional CYP450 genes that were not significant in the overall breed contrast, *CYP2B22* and *CYP2J34*, were significant for the pairwise breed contrast. Multiple CYP450 genes displayed significant sex contrast differences for FBZ (*CYP1A1* and *CYP2B22*) and FLU (*CYP2B22*, *CYP2C36* and *CYP7A1*). Lastly, a small proportion of the genes were confirmed using qPCR and the results are outlined in Figures S4 and S5. Across all 7 genes tested, the fold change values derived from quantitative real-time PCR (qPCR) utilizing the full dataset were similar to the fold change values estimated utilizing the RNA sequence data generated on the subset of the animals.

### Gene network (i.e. module) identification

**Figure 3:**
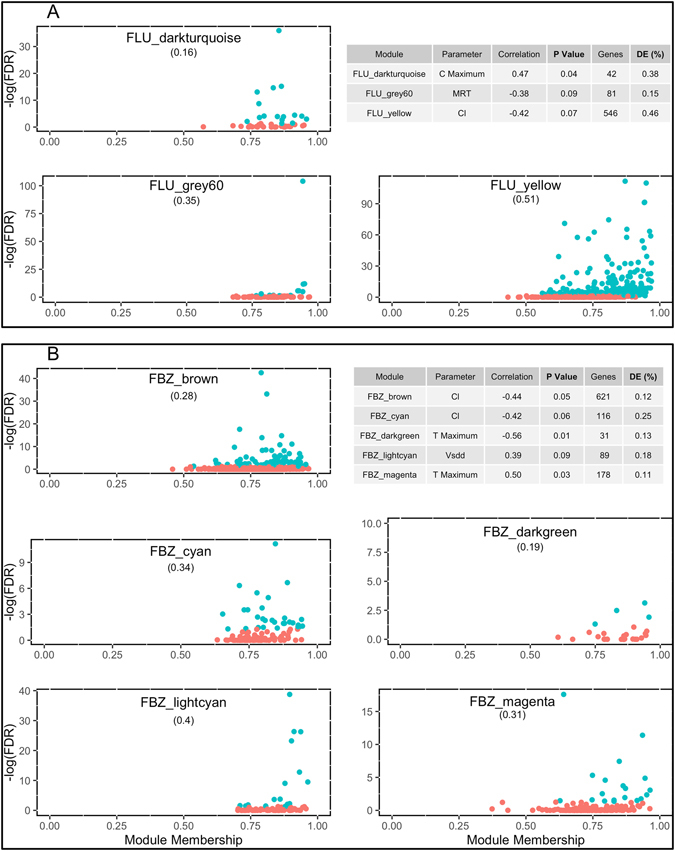
Significant modules associated with Flunixin Meglumin (i.e. Panel A) or Fenbendazole (Panel B) and the number of significant differentially expressed genes within each module.

A weighted gene co-expression network (WGCNA) and differential transcript analysis was conducted using the count data generated from RNA-Seq. These two approaches were utilized in tandem in order to identify a subset of genes that impacted how an animal metabolized the drug based on its relationship with a PK parameter and displayed differences in transcript levels between animals administered FBZ or FLU versus UNT animals. The count data generated from RNA-Seq was transformed to reads per kilobase of transcripts per million-mapped (RPKM) and normalized, log2 transformed and adjusted for the batch effects prior to the analysis. Similar to the gene filtering used in the differential transcript analysis, genes with a low transcript count were removed and the remaining genes were normalized, which resulted in a total of 11,877 genes within each analysis. A subset of genes, referred to as modules, with correlated expression levels were generated within animals administered FLU and FBZ using the WGCNA package^12^ (version 1.42). All modules were assigned a color that will be hereinafter used to reference the modules and is depicted in the hierarchical clustering dendogram outlined in Figure S6. To determine if any of the modules were associated with a PK parameter, the correlation between module eigengenes (defined as the first principal component of a given module and represents the gene expression profiles in the given module) and each PK parameter were estimated across all modules and results across modules and PK parameters are outlined in Figures S7 and S8 for FBZ and FLU, respectively. Three and five modules were significantly correlated (|correlation| > 0.30 & P-value < 0.10) with a PK parameter and at least 10% of the genes displayed differential transcript levels for FLU and FBZ, respectively. Relationships between the significance level of a gene based on the differential expression analysis and module membership (MM; defined as a measure of the strength of the gene belonging to the module) along with descriptive statistics for each significant module are outlined in Fig. 3. For the majority of the modules, genes with significant (FDR < 0.05) transcript differences between animals given a drug versus untreated were the most important genes (i.e. high MM value) in the module.

**Figure 4:**
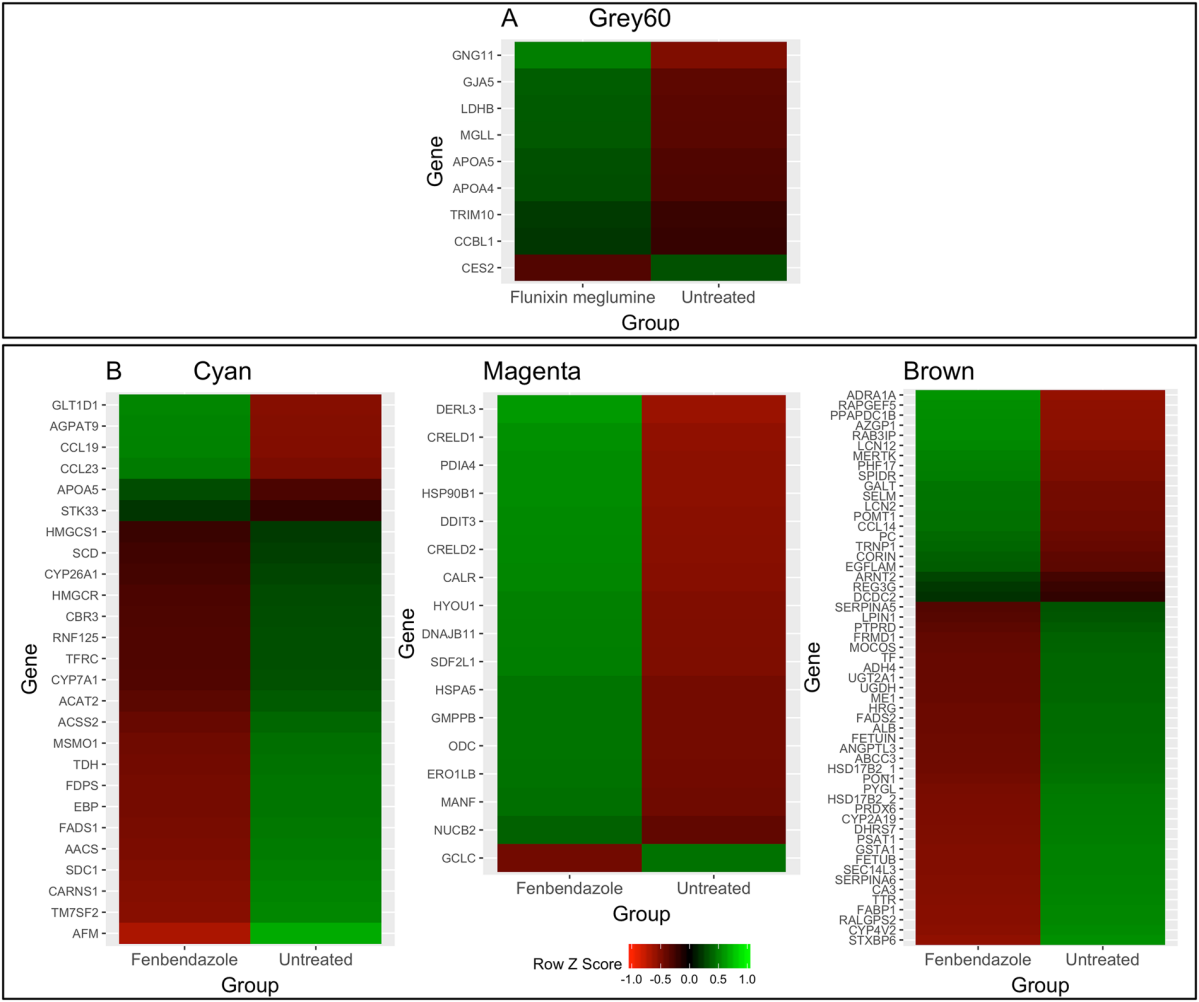
Expression profiles of genes with significant differences in transcript levels across animals administered Flunixin Meglumine (Panel A) and Fenbendazole (Panel B) versus untreated animals within modules associated with pharmacokinetic phenotypes.

An enrichment analysis was conducted within each of the modules declared significant. For FBZ, three out of the five modules were enriched with GO annotations terms, while only one GO annotation was enriched for FLU and is reported in Table 1. The Cyan module was negatively correlated (−0.42; P-value = 0.06) with the FBZ clearance PK parameter and was enriched for processes related to lipid metabolism (GO-ID: 0006629). The number of genes contained within the cyan module was 116 and 25% of these genes displayed significantly different transcript levels across treated versus untreated. The magenta module contained 178 genes and was positively correlated (0.50; P-value = 0.01) with the time at maximum concentration for the FBZ metabolite (oxfendazole) and was enriched for response to endoplasmic reticulum stress (GO-ID: 0034976). Out of the 178 genes within the magenta module, 11% of the genes had different transcript levels across treated versus untreated. The brown module contained a large number of genes (n = 621) and 12% displayed significant different transcript levels across treated versus untreated. The brown module was negatively correlated (−0.44; P-value = 0.05) with the FBZ clearance PK parameter and was enriched for processes related to small molecule metabolism (GO-ID: 0044281). Lastly, the grey60 module was negatively associated (−0.38; P-value = 0.04) with the mean residence time of FLU and was enriched for carboxylic acid metabolic processes (GO-ID: 0019752). The number of genes contained within the grey60 module was 81 and 15% of the genes displayed significantly different transcript levels across treated versus untreated. Expression profiles of genes with known GO-annotations and significant transcript differences across treated and untreated are outlined in Fig. 4. A heat map illustrating the correlation between normalized and batch-adjusted RPKM values across genes within the cyan, brown and magenta module for FBZ and grey60 module for FLU is shown in Figures S9, S10, S11 and S12, respectively.

**Table 1:** Overview of the most significant overrepresented GO terms associated with the modules using WGCNA.

Drug	WGCNA Module	GO-term	Number of genes with GO term in Module	Number of genes in term	False DiscoveryRate (FDR)
Fenbendazole	Cyan	Lipid Metabolic Process	18	330	1.65E-04
	Magenta	Response to Endoplasmic Reticulum Stress	15	80	3.24E-07
	Brown	Small Molecule Metabolic Process	62	456	2.19E-06
Flunixin	Grey60	Carboxylic Acid Metabolic Process	11	243	2.80E-02

### Differences in connectivity between animals administered a drug versus untreated animals

The connectivity of a gene is a measure of gene interactions between a given gene and all genes within the network. Furthermore, the previously described modules consist of a group of highly interconnected genes, and as a consequence of scale-free topology assumptions, will consist of many lowly interconnected genes and a few highly interconnected genes that are referred to as hub genes^13^. Three networks were constructed based on subsets of the RNA-Seq data and include animals administered FBZ or FLU and UNT. In order to identify hub genes within the previously defined significant modules, the difference in scaled connectivity between FBZ versus UNT and FLU versus UNT were estimated. Genes were investigated further if their absolute connectivity was greater than 0.6. When the differences between connectivity values was positive, it meant that genes were more highly connected in the treated group than in the untreated group. Conversely, a negative connectivity value designated genes that were more connected in the untreated group than in the treated group. Within the cyan, magenta and brown modules, 3 (*CYP51*, *MSMO1* and *SQLE)*, 3 (*LOC100513955*, *LOC100525076*, *STX5*) and 6 (*H3F3A*, *HADH*, *HAO1*, *LOC100738422*, *OTC* and *SLC31A1*) hub genes were identified for FBZ, respectively. Furthermore, all connectivity values within the genes were positive meaning they were more connected in the treated than in the untreated group. Within the FLU grey60 module, 4 genes displayed positive connectivity values (*ECH1*, *ICAM2*, *LOC100520636* and *PFKL*) and 2 genes displayed negative connectivity values (*LOC100516466* and *LOC100739849*). None of the declared hub genes displayed significant (FDR < 0.05) transcript differences. Expression profiles of hub genes across treated and untreated are summarized in Figure S13.

**Figure 5:**
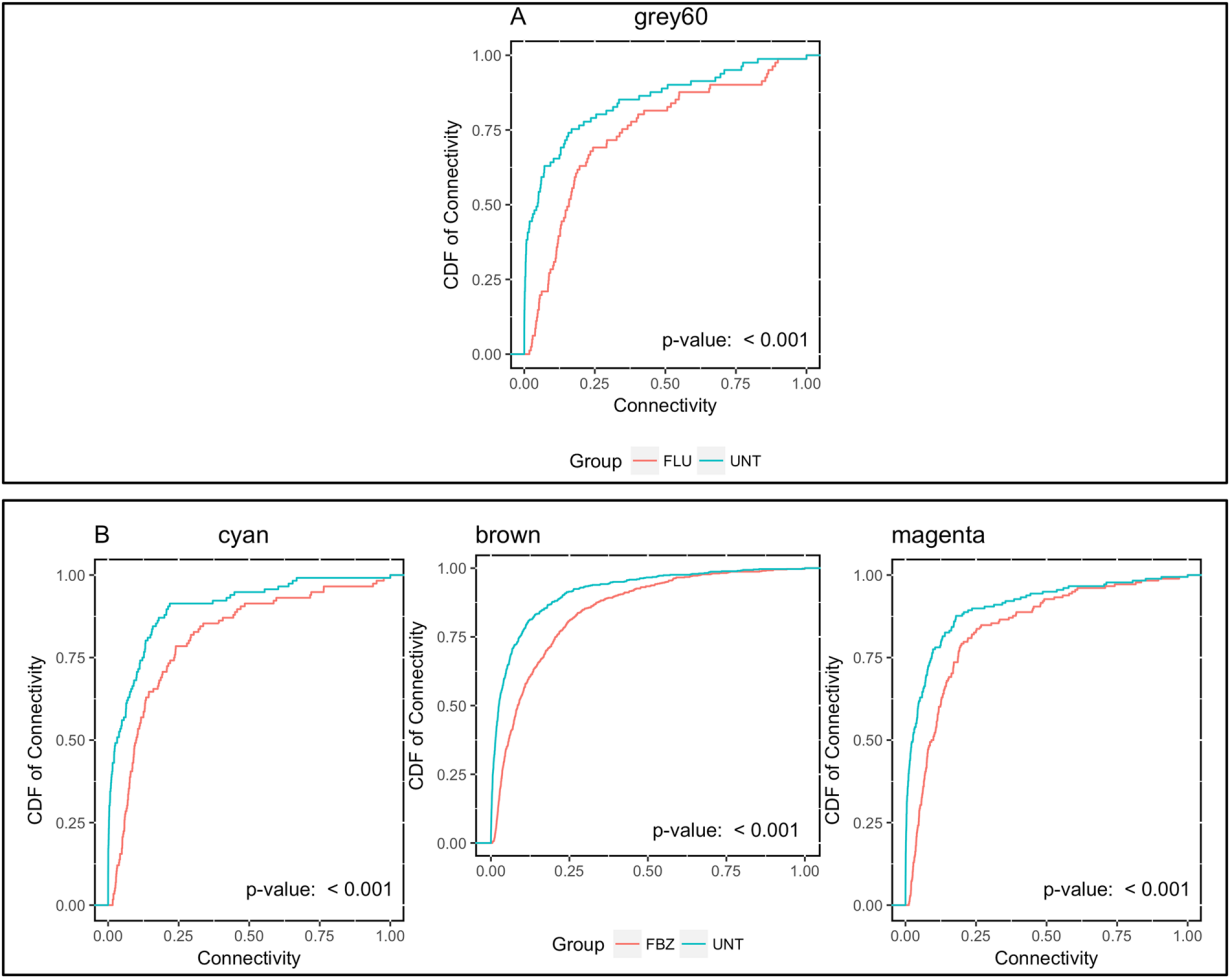
Cumulative density function (CDF) of differential connectivity values between animals administered Flunixin Meglumin (FLU; Panel A) and Fenbendazole (FBZ; Panel B) versus untreated (UNT) animals within modules associated with pharmacokinetic phenotypes and the significance based on the Kolmogrov-Smirnov test.

Lastly, in order to determine whether genes display similar connectivity values within the treated versus untreated groups, the distribution of differential connectivity values for genes within significant modules were compared between treated versus untreated. Differences between the distributions of the connectivity values between treated versus untreated were tested for significance using the non-parametric Kolmogrov-Smirnov test. The mean connectivity within genes for modules associated with PK parameters was always greater in the treated versus the controls as outlined in Fig. 5. Therefore, given the highly significant Kolmogrov-Smirnov test statistic (P-value < 0.001) the genes within a given module appear to have a higher degree of correlation in the presence of either drug compared to the untreated animals.

## Discussion

The current study utilized RNA sequence data to better understand how the liver transcriptome is impacted by FBZ or FLU administration and identify individual gene and gene networks that impact drug metabolism and clearance. The identification of genetic determinants and/or biological pathways that give rise to individual variability in drug efficacy and safety is a major challenge in the context of human medicine regarding current clinical practice, drug development and drug regulation^14^. Furthermore, in the context of animal derived foods, a recent article addressed the issues relating to establishing drug tolerance and withdrawal times for unhealthy animals and its inability to address the variation across animals and how disease status impact drug clearance^4^. Therefore, the identification of genes or sets of genes that result in variation in drug response not only has impacts on being able to more effectively administer drugs to livestock, but also to gain a better understanding of gene networks that are involved in drug metabolism.

The current study found multiple cytochrome P450 genes differentially expressed across treated versus untreated individuals in addition to evidence for differences in transcript counts within breed and sex. The *CYP1*, *CYP2* and *CYP3* gene families are primarily involved in xenobiotic metabolism and display selective but overlapping substrate specificity so that they can metabolize a large array of chemicals^15^. The expression of CYP450 gene products are influenced by a unique combination of mechanisms and factors including genetic polymorphisms, induction by xenobiotics, regulation by cytokines, hormones, sex and disease states^16^. Furthermore, multiple genetic variants have been found within the CYP450 family to impact drug metabolism and clearance and a review is outlined in Zanger & Schwab^16^. Across both FBZ and FLU, multiple genes within the *CYP1*, *CYP2* and *CYP3* gene families were found to have different transcript levels across treated individuals versus treated and they include *CYP1A1*, *CYP2A19* and *CYP2C36*. The majority of CYP450 genes were suppressed when drugs were administered with the exception of *CYP2C36*. Furthermore, the *CYP2B22* gene displayed differences in transcript levels across breed and sex for both FBZ and FLU. Previous work by Howard *et al*.^7^ found the *CYP1A2* to be significantly different across animals administered a drug versus untreated utilizing the same dataset, although the fold change was in the opposite direction. The dataset utilized in the RNA-seq study was a subset of the complete data set and therefore this may result in different fold change values due to the use of a smaller dataset.

Multiple modules were identified that were correlated with PK parameters across both FBZ and FLU. For FBZ, three out of the five modules were enriched with GO annotation terms. The Cyan module was enriched for processes related to lipid metabolism and negatively correlated (−0.42) with the clearance PK parameter. Altered lipid metabolism levels across individuals can give rise to altered lipid profiles within the body and altered CYP450 expression levels, which would then impact the bioavailability of the drug and the proportion bound in the plasma^17, 18^. Genes that were differentially expressed within the lipid metabolism annotation term were associated with cholesterol and bile acid metabolism and included *AACS*, *ACAT2, ACSS2*, *CYP7A1*, *APOA5*, *FDPS*, *TM7SF2*, *MSMO1*, *HMGCS1 and EBP*. Bile acids have been shown to be important regulators in drug disposition^19^ and the *CYP7A1* gene is the initial and rate limiting step in the catabolism of cholesterol to bile acids^20^. Previous research has shown that the level of bile acids can alter the blood concentrations across multiple lipophilic drugs when given orally, which therefore impact the pharmacokinetic parameters of the drug^21–23^. Furthermore, fenbendazole is eliminated through bile into feces in rats and mice^24^ and therefore any alterations in the level of bile salts could alter clearance of the drug. The impact of bile acid is likely to be influenced to a greater degree when administered orally instead of intravenously, which was the route utilized in this study. However, if enterohepatic recirculation occurs, which has been shown to occur in sheep^25^, bile acids may play a role in metabolizing FBZ.

The magenta module was enriched for processes related to endoplasmic reticulum (ER) stress and positively correlated (0.50) with the time at maximum concentration for the FBZ metabolite, oxfendazole. Gene expression profiles within the module that had different transcript levels across treated versus untreated are outlined in Fig. 4 and illustrate the upregulation of these genes in the liver of animals administered FBZ. Accumulation of unfolded proteins in the ER induces stress and is a cellular mechanism that aids in protecting the ER, first by recruiting ER-resident stress proteins and, if conditions don’t improve, the apoptotic response process is initiated^26^. The induction of ER stress due to FBZ administration has been found in cancer cell lines^27^ and prolonged oxfendazole administration has been shown to induce apoptosis in meiotic spermatocytes and altered ER in the Sertoli cells in rats^28^. Some of the major genes in the ER stress pathway, as outlined in Chow *et al*.^29^, were differentially expressed across treated versus untreated and include *DDIT3*, *HSPA5* and *HYOU1*.

The Brown module was enriched for processes related to small molecule metabolism and was negatively correlated (−0.44) with the FBZ clearance. There were a large number of genes (n = 62) within the enriched annotation term and therefore only the ones with a high module membership and/or significant transcript differences across treated versus untreated will be discussed. Most drugs, including FBZ and FLU, are classified as small molecules and therefore processes related to small molecule metabolism should be impacted following drug administration. The UGDH gene product is critical in hepatic tissue for endoplasmic reticulum-localized Phase II detoxification of lipophilic hormones and xenobiotics by glucuronidation^30^. Furthermore, multiple genes were within the module with processes related to phase I drug metabolism including dehydrogenases (*ADH4*, *ALDH5A1* and *HSD17B4*) and flavin containing monooxygenases (*FMO1*).

Lastly, the grey60 module was enriched for processes related to carboxylic acid metabolism and was negatively associated (−0.38) with the mean residence time of FLU. Flunixin meglumine is a non-steroidal anti-inflammatory drug (NSAID) and, like many NSAIDs, is a carboxylic acid-containing compound. The carboxylic acid moiety of FLU undergoes conjugation prior to being eliminated in the urine. Furthermore, FLU mediates its effects through inhibition of the cyclooxygenase enzyme and has been previously found to be impacted by cholesterol levels^31^. Two genes were included in this module related to cholesterol levels in the plasma (*SREBF1*, *APOA5*). Gene expression profiles within the module that had different transcript levels across treated versus untreated are outlined in Fig. 4, which illustrate the up-regulation of these genes within the group given FLU.

The connectivity of genes within each module was also computed separately for the treated and untreated animals in order to determine if the genes displayed a higher degree of connectivity in the treated versus untreated animals. It is hypothesized that upon drug administration the interaction among genes within a module should increase over that of an untreated module. Across all modules genes within the modules displayed a higher connectivity value than the untreated gene connectivity values. Furthermore, candidate hub genes within each module were identified based on the differential connectivity estimate. For the cyan module, which was enriched with lipid metabolism genes, 3 genes with functions related to cholesterol metabolism were identified (*CYP51*, *MSMO1* and *SQLE*). A hub gene within the magenta module, *STX5*, has been shown to play an important role in the ER stress response that precedes apoptotic cell death and has been shown to regulate the targeting and fusion of carrier vesicles within the ER^32^. The candidate hub genes identified in the current study and the increased connectivity with the treated group for the modules identified will need to be validated in other populations.

The current study has integrated gene co-expression networks, gene versus drug metabolizing parameter correlations and differential transcript analysis across animals administered FBZ or FLU versus UNT to provide new insights into individual gene and gene networks that impact drug metabolism. Alterations in the transcript profile within the liver following FBZ and FLU administration were seen. More importantly, alterations within a specific set of genes contained within identified modules were seen following drug administration and these modules were significantly correlated with drug PK parameters. Further investigation into genes that had significant transcript differences and genes contained within modules enriched with biologically relevant functions will allow for greater insight into how FBZ and FLU are metabolized and may lead to more effective treatments for livestock and humans.

## Methods

### Animals

This study was approved by the NCSU Institutional Animal Care and Use Committee (IACUC) and all experiments were performed in accordance with relevant guidelines and regulations. The samples and phenotypes utilized were derived from a population of animals generated to investigate the phenotypic and genetic variability related to FBZ and FLU drug metabolism and is described in detail by Howard *et al*.^2, 7^. Briefly, the animals in the full dataset were crossbred nursery females and castrated male pigs spread across 5 batches. The animals were split into batches due to insufficient pen space for all the animals. Within each batch animals were randomly placed into UNT, FLU or FBZ. The sires of the pigs were comprised of 4 separate breeds and were registered National Swine Registry boars mated to a common sow population at the North Carolina State University Swine Education Unit. The drugs, FBZ or FLU, were administered via IV administration in order to remove inter- and intra-individual variability often observed with extravascular routes of administration^9, 33^. An initial dose (FBZ dose: 1 mg/kg; FLU dose: 3 mg/kg) was given in order to collect blood samples across a 48-hr period, which was then utilized to estimate PK parameters. A non-compartmental analysis of drug and metabolite plasma concentration versus time profiles was performed with PK modeling Phoenix^®^ software (version 1.1; Pharsight, Cary NC, USA), as outlined previously by Howard *et al*.^2^. Parameters derived from PK modeling included the area under the plasma concentration–time curve from time zero to infinity (AUC_0→∞_; h*μg/mL), clearance (Cl; L/h/kg), half-life (T_1/2_; h), mean residence time (MRT; h), the volume of distribution at steady state (Vd_ss_; L/kg), peak concentration (C_max_; μg/mL) and time at which maximum concentration occurs (T_max_; h). After 48 h a second dose was given and, one hour after drug administration, animals were sacrificed via captive bolt and a liver sample from the right lateral lobe was collected and placed in RNA Later (Qiagen, Valencia, CA, USA). A liver collection sample of 1 hour was chosen based on a compromise between the two drugs on the maximal levels of metabolite and drug levels in the plasma and is the concentration verse time plots are outlined in Howard *et al*.^7^. Previous work by Howard *et al*.^2^ has shown that negligible amounts of the drug and metabolite remained in the blood plasma after 48 hours and therefore the carry over effect from the first dose should be minimal.

### Experimental Design

The animals in the current study were selected from the full dataset with the aim to maximize the variability across animals in whole genome transcript levels that were related to drug metabolism. This was accomplished by selecting treated animals that were at the tails of the distribution for the clearance PK parameter within each. The untreated animals were selected randomly and balanced across breed, sex and batch, because PK parameter estimates were not available in these individuals. The majority of the effects are balanced across treatment groups and breed, although the sex class is slightly unbalanced for FLU, which was due to insufficient numbers of females within the Yorkshire breed so that an additional male was chosen instead. The number of sires within each breed class was chosen to be as uniform as possible in order reduce the effect of a breed contrast being influenced by a sire effect. Lastly, not all breeds were represented within each batch.

### RNA-Sequencing data, editing and normalization

The RNA-Seq samples were prepared following the manufacturer’s recommendation using the TruSeq RNA Sample Prep (Illumina, Hayward, CA, USA). Samples were sequenced on the Iseq platform, dividing the 60 samples across 7 lanes (i.e. 4 lanes with 9 samples and 3 lanes with 8 samples). Prior to alignment, sequence reads were trimmed to remove possible adapter sequences and nucleotides with poor quality (error rate < 0.5). After trimming, sequence reads shorter than 30 nucleotides were discarded. Remaining reads were aligned to the pig reference genome sequence assembly^34^ (Sscrofa10.2) using the CLC Genomics Server program (Qiagen, Valencia, CA, USA). The total number of uniquely mapped reads per sample was 22,260,237 and on average across samples, 17,683,594 were mapped.

### Differential Expression and Co-Expression Network Analysis

In order to ensure that genes associated with drug metabolism at PK levels were identified, a gene co-expression network analysis was conducted along with a differential transcript analysis. The total number of genes prior to any data editing across the 60 samples was 35,252. The data editing and normalization procedure was done using the Bioconductor package edgeR^11^ (version 3.8.6). The read counts per gene were normalized to counts per million (CPM). Previous studies have shown that genes with low expression levels are less reliable^13, 35^, therefore genes with CPM values above 1 in at least 5 samples were kept. The final number of genes utilized across all analysis was 11,877. Furthermore, in order to account for the compositional differences between the libraries, CPM values were normalized based on the trimmed mean of M-values normalization procedure. Transcript levels were also expressed as RPKM and determined for each gene and sample using the *rpkm* function within the edgeR package^11^ (version 3.8.6). The differential transcript analysis used the CPM values and the gene co-expression network used the RPKM values. The RPKM value was used for the gene co-expression network to minimize the effect of gene length bias when relating expression levels across genes, whereby longer genes will be sequenced deeper than shorter genes^36^. Within each drug, clustering of samples by transcript count (transformed to log2 CPM) was evaluated for the animals given the particular drug and untreated animals across batches using the *plotMDS* function in the Bioconductor package limma^37^ (version 3.22.7). This function uses 500 genes that have the largest standard deviation transcript levels between samples to construct the Euclidian distance between each pair of samples.

Differential transcript analysis on the 11,877 genes remaining after gene filtering was conducted using edgeR^11^ (version 3.8.6). Two separate analyses were conducted to identify across breed and sex transcript differences and within breed or sex differences. The first stage was conducted to identify genes that had different transcript levels across treated versus untreated animals utilizing normalized CPM values based on a model that accounted for the batch effect. The second stage of the analysis identified genes with transcript levels that differed across breed and sex after drug administration using normalized CPM values. The following contrasts were used for each pairwise breed contrast within a drug:

$$(Treate{d}_{BreedA}-Untreate{d}_{BreedA})-(Treate{d}_{BreedB}-Untreate{d}_{BreedB}),$$

and the sex contrast within each drug:

$$(Treate{d}_{Male}-Untreate{d}_{Male})-(Treate{d}_{Female}-Untreate{d}_{Female}).$$

The effect of batch was accounted for in the breed and sex contrasts. Genes were investigated if they had FDR values less than 0.05.

Pigs administered either FBZ or FLU were utilized to identify gene co-expression networks correlated with PK phenotypes. Furthermore, modules were checked to determine the frequency of genes that had significant differences in CPM values based on the previous analysis in order to ensure biologically relevant modules were identified. Prior to the analysis, log transformed RPKM values were corrected for the group effect using the *removeBatchEffect* function from the Bioconductor package limma^37^ (version 3.22.7). An un-signed co-expression network was built using the WGCNA package^12^ (version 1.42). An adjacency matrix within each drug was created by calculating the Pearson’s correlations between all genes that remained after quality control and raised to a soft-threshold power (β) of 18. The power β was chosen utilizing the pickSoftThreshold() function within the WGCNA package^12^, which is based on the scale-free topology criterion^38^. The average scale-free topology index (R^2^) was 0.91 and 0.89 for FBZ and FLU, respectively. To identify modules of co-expressed genes, a topological overlap-based dissimilarity matrix^38, 39^ (TOM) was constructed using the adjacency matrix. The dissimilarity TOM was used as input for the average linkage hierarchical clustering algorithm, which resulted in a clustering tree (dendrogram). Clusters of highly interconnected genes (i.e., set of genes co-expressed), referred to as modules, were determined as branches of the dendrogram using the Dynamic Tree-Cut algorithm^40^. Furthermore, the module eigengene was used to represent each module and was calculated by the first principal component in order to capture the maximal amount of variation of each module. Modules whose eigengenes were highly correlated (r > 0.85) were further merged into a single module. The aforementioned steps were completed using the one-step network construction and module detection function within the WGCNA package^12^ (version 1.42) and the following major parameters: maxBlockSize of 15000, minModuleSize of 30, reassignThreshold of 0 and mergeCutHeight of 0.15.

After modules were constructed within each drug, correlations between module eigengenes and PK parameters were estimated. Modules whose correlations were greater in absolute value than 0.30 and a significance value less than 0.10 were kept and investigated further^41^. The final step prior to gene annotation was to only keep modules that had at least 10 percent of the genes displaying significant (FDR < 0.05) transcript differences across animals administered a drug versus untreated animals. The previous steps were utilized to select potential biologically relevant modules that displayed transcript level variability across samples for downstream analyses.

Genes of interest within the modules were prioritized based on the strength of a gene’s MM, connectivity value and significance based on the previous differential expression analysis results. The connectivity (measure of gene interaction with other genes) of all genes within modules passing the previous threshold was determined using the *softconnectivity* function within the WGCA package^12^ (version 1.42). The MM parameter is a measure of the strength of a particular gene’s membership in a module obtained by correlating the gene’s expression profile with the module eigengene of that given module^41^. Genes within modules that passed the previous steps were annotated using the Bioconductor package biomaRt^42, 43^ (version 2.22.0). Gene ontology^44^ (GO) term enrichment tests were performed for each individual module and compared to a background set of all genes expressed in the liver using the Blast2Go software^45^. Significance was declared for any GO term that had a FDR value of less than 0.05. Lastly, multiple genes were chosen based on their differential expression, module membership and differential connectivity to be confirmed in the full dataset (n = 69 FBZ; n = 72 FLU; n = 40 UNT) using qPCR across both FBZ and FLU. The genes included *ABCC3*, *CYP2A19*, *CYP4V2*, *CYP7A1*, *GSTA1*, *LCN2* and *CA3*. A linear mixed model that has a similar framework as Steibel *et al*.^46^ and is described in detail by Howard *et al*.^7^ was utilized and contrasts similar to the pairwise breed contrasts in the differential expression analysis were constructed. Significance was adjusted using the Bonferroni correction due to multiple breed/sex comparisons.

The connectivity of a gene is a measure of gene interactions, in the form of co-expression, between a given gene and all genes within the network. Furthermore, depending on the state of the phenotype, treated versus untreated, the connectivity of a gene may be altered and, biologically, describes different active pathways in treated versus untreated animals. Within each module, an adjacency matrix was computed for the respective network (i.e. FLU or FBZ treatment group), as described previously in the WGNCA section, and for each gene the connectivity value was estimated. Only genes that were contained within modules of interest were investigated. The connectivity value is the sum of the adjacency values to all other genes within the module and is divided by the maximum connectivity value within the network, which constrains the range of values between 0 and 1. In order to identify hub genes, the difference in the connectivity value for the treated and untreated network for each gene was estimated. Genes were investigated further if their absolute connectivity was greater than 0.6^13^. In order to determine whether genes display higher connectivity values within the group administered a drug versus untreated individuals, networks were constructed for treated (i.e. FBZ or FLU) and untreated within each module. The distribution of differential connectivity values were compared within each module across treated (i.e. FBZ or FLU) and untreated. Differences between the distributions of the connectivity values within each module between treated versus untreated were tested for significance using the non-parametric Kolmogrov-Smirnov test.

## Electronic supplementary material

Supplementary Information

## References

[1] Landers TF, Cohen B, Wittum TE, Larson EL (2012). A review of antibiotic use in food animals: perspective, policy, and potential. Public Health Rep. Wash. DC 1974.

[2] Howard JT (2014). The effect of breed and sex on sulfamethazine, enrofloxacin, fenbendazole and flunixin meglumine pharmacokinetic parameters in swine. J. Vet. Pharmacol. Ther..

[3] Kissell LW, Leavens TL, Baynes RE, Riviere JE, Smith GW (2015). Comparison of pharmacokinetics and milk elimination of flunixin in healthy cows and cows with mastitis. J. Am. Vet. Med. Assoc..

[4] Lin Z, Vahl CI, Riviere JE (2016). Human Food Safety Implications of Variation in Food Animal Drug Metabolism. Sci. Rep.

[5] Baynes RE (2016). Health concerns and management of select veterinary drug residues. Food Chem. Toxicol. Int. J. Publ. Br. Ind. Biol. Res. Assoc.

[6] Lin Z, Lou Y, Squires EJ (2006). Functional polymorphism in porcine CYP2E1 gene: Its association with skatole levels. J. Steroid Biochem. Mol. Biol..

[7] Howard JT, O’Nan AT, Maltecca C, Baynes RE, Ashwell MS (2015). Differential Gene Expression across Breed and Sex in Commercial Pigs Administered Fenbendazole and Flunixin Meglumine. PloS One.

[8] Bendixen E, Danielsen M, Larsen K, Bendixen C (2010). Advances in porcine genomics and proteomics-a toolbox for developing the pig as a model organism for molecular biomedical research. Brief. Funct. Genomics.

[9] Petersen MB, Friis C (2000). Pharmacokinetics of fenbendazole following intravenous and oral administration to pigs. Am. J. Vet. Res..

[10] Königsson K, Törneke K, Engeland IV, Odensvik K, Kindahl H (2003). Pharmacokinetics and pharmacodynamic effects of flunixin after intravenous, intramuscular and oral administration to dairy goats. Acta Vet. Scand..

[11] Robinson MD, McCarthy DJ, Smyth GK (2010). edgeR: a Bioconductor package for differential expression analysis of digital gene expression data. Bioinforma. Oxf. Engl.

[12] Langfelder P, Horvath S (2008). WGCNA: an R package for weighted correlation network analysis. BMC Bioinformatics.

[13] Kogelman LJA (2014). Identification of co-expression gene networks, regulatory genes and pathways for obesity based on adipose tissue RNA Sequencing in a porcine model. BMC Med. Genomics.

[14] Ma Q, Lu AYH (2011). Pharmacogenetics, pharmacogenomics, and individualized medicine. Pharmacol. Rev..

[15] Porter TD, Coon MJ (1991). Cytochrome P-450. Multiplicity of isoforms, substrates, and catalytic and regulatory mechanisms. J. Biol. Chem.

[16] Zanger UM, Schwab M (2013). Cytochrome P450 enzymes in drug metabolism: regulation of gene expression, enzyme activities, and impact of genetic variation. Pharmacol. Ther..

[17] Wasan KM, Brocks DR, Lee SD, Sachs-Barrable K, Thornton SJ (2008). Impact of lipoproteins on the biological activity and disposition of hydrophobic drugs: implications for drug discovery. Nat. Rev. Drug Discov..

[18] Pikuleva IA (2006). Cytochrome P450s and cholesterol homeostasis. Pharmacol. Ther..

[19] Eloranta JJ, Kullak-Ublick GA (2005). Coordinate transcriptional regulation of bile acid homeostasis and drug metabolism. Arch. Biochem. Biophys..

[20] Pandak WM (2001). Effects of CYP7A1 overexpression on cholesterol and bile acid homeostasis. Am. J. Physiol. Gastrointest. Liver Physiol..

[21] Gutzler F, Zimmermann R, Ring GH, Sauer P, Stiehl A (1992). Ursodeoxycholic acid enhances the absorption of cyclosporine in a heart transplant patient with short bowel syndrome. Transplant. Proc..

[22] Lindholm A, Henricsson S, Dahlqvist R (1990). The effect of food and bile acid administration on the relative bioavailability of cyclosporin. Br. J. Clin. Pharmacol..

[23] Sasaki M, Maeda A, Sakamoto K, Fujimura A (2001). Effect of bile acids on absorption of nitrendipine in healthy subjects. Br. J. Clin. Pharmacol.

[24] Villar D, Cray C, Zaias J, Altman NH (2007). Biologic effects of fenbendazole in rats and mice: a review. J. Am. Assoc. Lab. Anim. Sci. JAALAS.

[25] Hennessy DR, Steel JW, Prichard RK (1993). Biliary secretion and enterohepatic recycling of fenbendazole metabolites in sheep. J. Vet. Pharmacol. Ther..

[26] Tsutsumi S (2004). Endoplasmic reticulum stress response is involved in nonsteroidal anti-inflammatory drug-induced apoptosis. Cell Death Differ.

[27] Dogra N, Mukhopadhyay T (2012). Impairment of the ubiquitin-proteasome pathway by methyl N-(6-phenylsulfanyl-1H-benzimidazol-2-yl)carbamate leads to a potent cytotoxic effect in tumor cells: a novel antiproliferative agent with a potential therapeutic implication. J. Biol. Chem..

[28] Okamura M, Watanabe T, Kashida Y, Machida N, Mitsumori K (2004). Possible mechanisms underlying the testicular toxicity of oxfendazole in rats. Toxicol. Pathol..

[29] Chow CY, Wang X, Riccardi D, Wolfner MF, Clark AG (2015). The genetic architecture of the genome-wide transcriptional response to ER stress in the mouse. PLoS Genet..

[30] Hyde AS, Thelen AM, Barycki JJ, Simpson MA (2013). UDP-glucose dehydrogenase activity and optimal downstream cellular function require dynamic reorganization at the dimer-dimer subunit interfaces. J. Biol. Chem..

[31] Smith LH, Petrie MS, Morrow JD, Oates JA, Vaughan DE (2005). The sterol response element binding protein regulates cyclooxygenase-2 gene expression in endothelial cells. J. Lipid Res..

[32] Suga K, Saito A, Akagawa K (2015). Data supporting ER stress response in NG108-15 cells involves upregulation of syntaxin 5 expression and reduced amyloid β peptide secretion. Data Brief.

[33] Pairis-Garcia MD (2013). Pharmacokinetics of flunixin meglumine in mature swine after intravenous, intramuscular and oral administration. BMC Vet. Res..

[34] Groenen MAM (2012). Analyses of pig genomes provide insight into porcine demography and evolution. Nature.

[35] Łabaj PP (2011). Characterization and improvement of RNA-Seq precision in quantitative transcript expression profiling. Bioinforma. Oxf. Engl.

[36] Oshlack A, Wakefield MJ (2009). Transcript length bias in RNA-seq data confounds systems biology. Biol. Direct.

[37] Ritchie ME (2015). limma powers differential expression analyses for RNA-sequencing and microarray studies. Nucleic Acids Res..

[38] Zhang B, Horvath S (2005). A general framework for weighted gene co-expression network analysis. Stat. Appl. Genet. Mol. Biol..

[39] Ravasz E, Somera AL, Mongru DA, Oltvai ZN, Barabási AL (2002). Hierarchical organization of modularity in metabolic networks. Science.

[40] Langfelder P, Zhang B, Horvath S (2008). Defining clusters from a hierarchical cluster tree: the Dynamic Tree Cut package for R. Bioinforma. Oxf. Engl.

[41] Kommadath A (2014). Gene co-expression network analysis identifies porcine genes associated with variation in Salmonella shedding. BMC Genomics.

[42] Durinck S (2005). BioMart and Bioconductor: a powerful link between biological databases and microarray data analysis. Bioinforma. Oxf. Engl.

[43] Durinck S, Spellman PT, Birney E, Huber W (2009). Mapping identifiers for the integration of genomic datasets with the R/Bioconductor package biomaRt. Nat. Protoc..

[44] Ashburner M (2000). Gene ontology: tool for the unification of biology. The Gene Ontology Consortium. Nat. Genet..

[45] Conesa A (2005). Blast2GO: a universal tool for annotation, visualization and analysis in functional genomics research. Bioinforma. Oxf. Engl.

[46] Steibel JP, Poletto R, Coussens PM, Rosa GJM (2009). A powerful and flexible linear mixed model framework for the analysis of relative quantification RT-PCR data. Genomics.

