# Supplementary material for: Gene co-expression network analysis identifies porcine genes associated with variation in metabolizing fenbendazole and flunixin meglumine in the liver
Source: Sci Rep. 2017 May 2;7:1357. doi: 10.1038/s41598-017-01526-5 (PMC5430975; doi:10.1038/s41598-017-01526-5)

Gene co-expression network analysis identifies porcine genes associated with variation in metabolizing fenbendazole and flunixin meglumine in the liver.

Jeremy T. Howard[1], Melissa S. Ashwell[1], Ronald E. Baynes[2], James D. Brooks[2], James L. Yeatts[2], Christian Maltecca[1,*].

[1]Department of Animal Science, North Carolina State University, Raleigh, NC 27695-7621, USA.

[2]Department of Population Health and Pathobiology, Center for Chemical Toxicology and Research Pharmacokinetics, North Carolina State University, College of Veterinary Medicine, 4700 Hillsborough Road, Raleigh, North Carolina 27606, USA.

Corresponding Author: Christian Maltecca

Address: Department of Animal Science

North Carolina State University,

Raleigh, NC 27695-7627

Phone: (919) 515-0812

Fax: (919) 515-6884

E-mail: christian_maltecca@ncsu.edu

Figure S1. Fenbendazole pharmacokinetic phenotypes for drug (i.e. FBZ) and metabolite (oxfendazole; OXF).

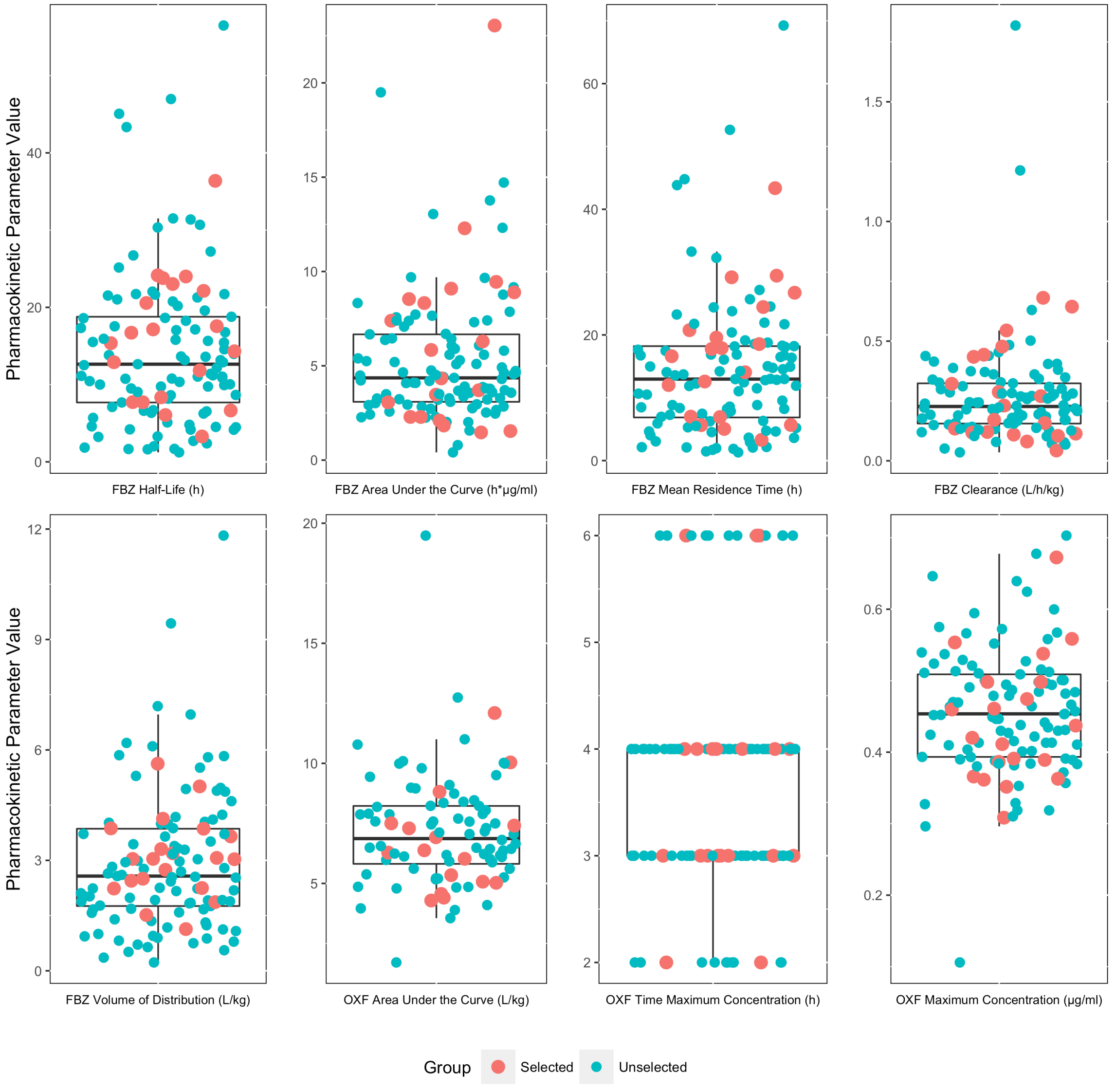

Figure S2. Flunixin meglumine pharmacokinetic parameters for drug (i.e. FLU) and metabolite (5-hydroxy flunixin; OH5).

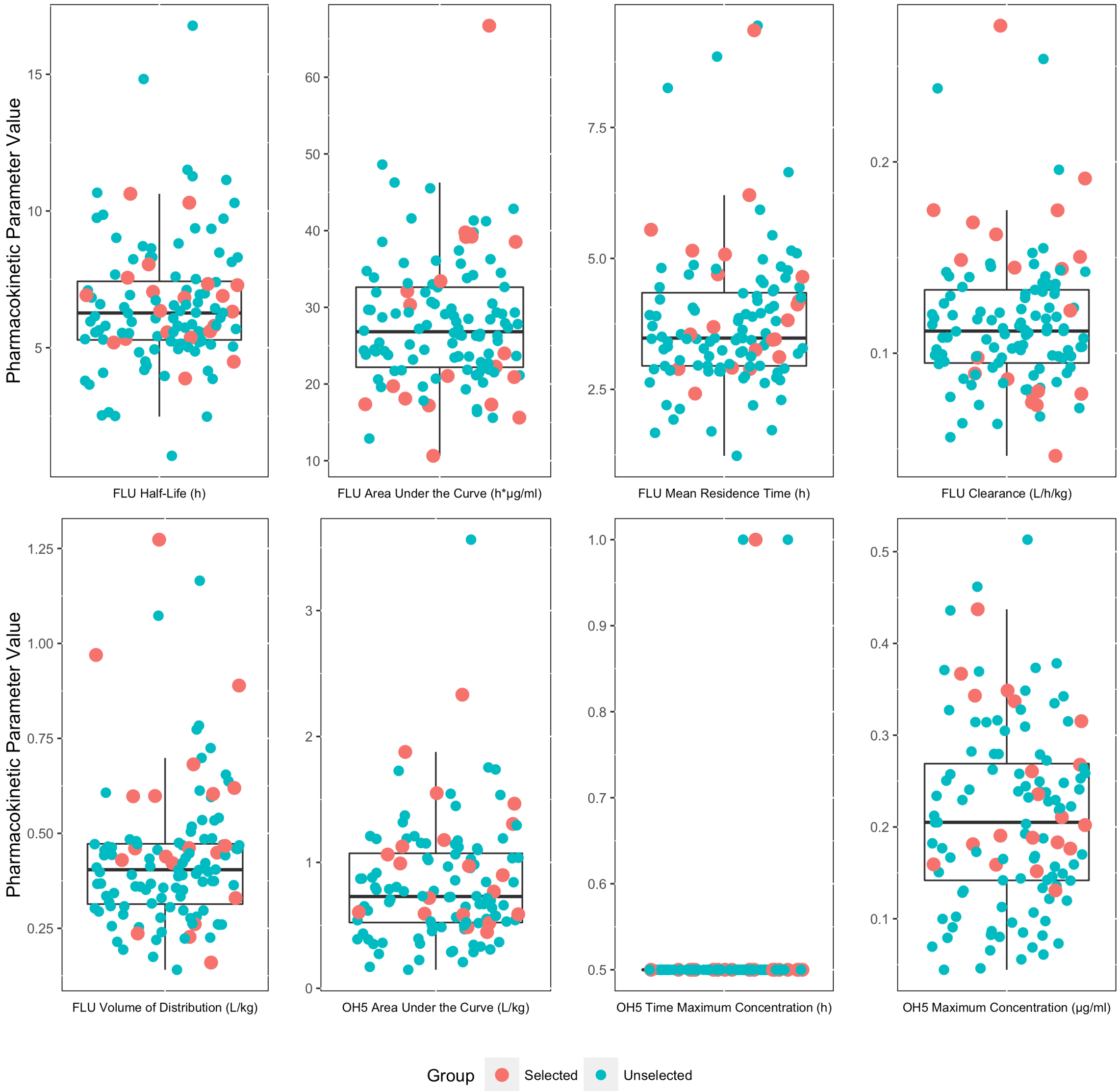

Figure S3. Multi-dimensional scaling plot of gene expression within each drug[1] against the control (UNT).

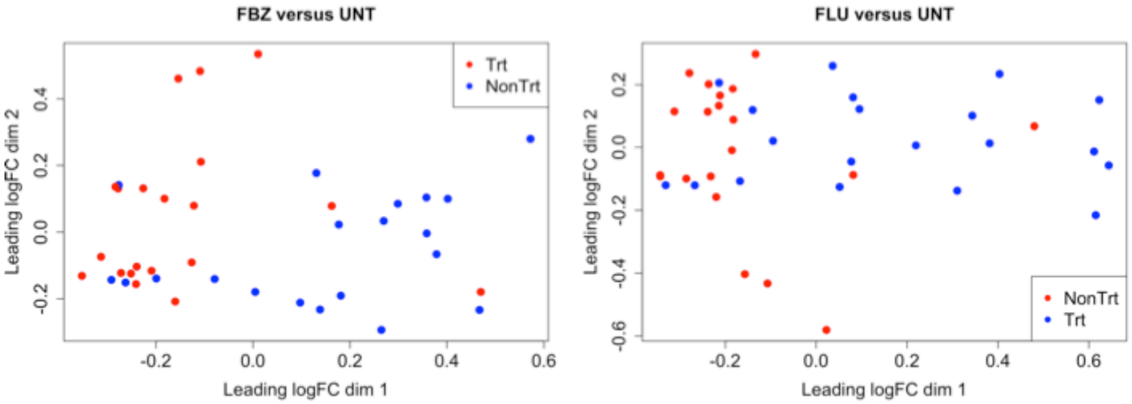

[1] FBZ refers to fenbendazole; FLU refers to flunixin meglumine

Figure S4. Fold change[1,2] between animals given fenbendazole and untreated animals across target genes compared to fold changes estimated from RNA-sequence data[3].

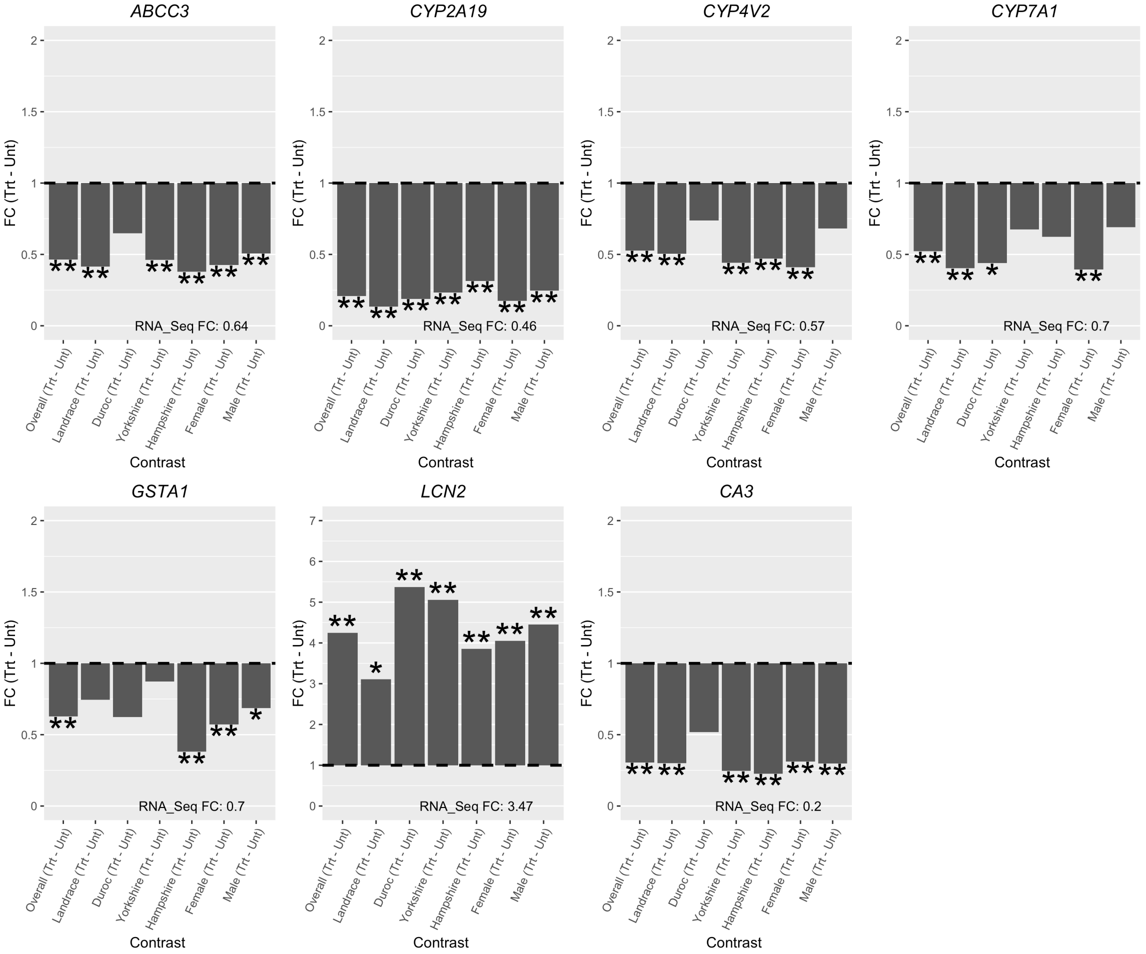

[1] The significance threshold was set at 0.0035 (i.e. **) and a tendency was set at 0.0071 (i.e. *) after the Bonferonni Correction.

[2] Genes up-regulated in treatment have a fold change > 1; Genes down-regulated in treatment fold change < 1.

[3] The RNA Sequence fold change is displayed in the bottom of the figure and the same contrast based on quantitative PCR utilizing the full dataset is the column within each plot (Overall (Trt – Unt)).

Figure S5. Fold change[1,2] between animals given flunixin meglumine and untreated animals across target genes compared to fold changes estimated from RNA-sequence data[3].

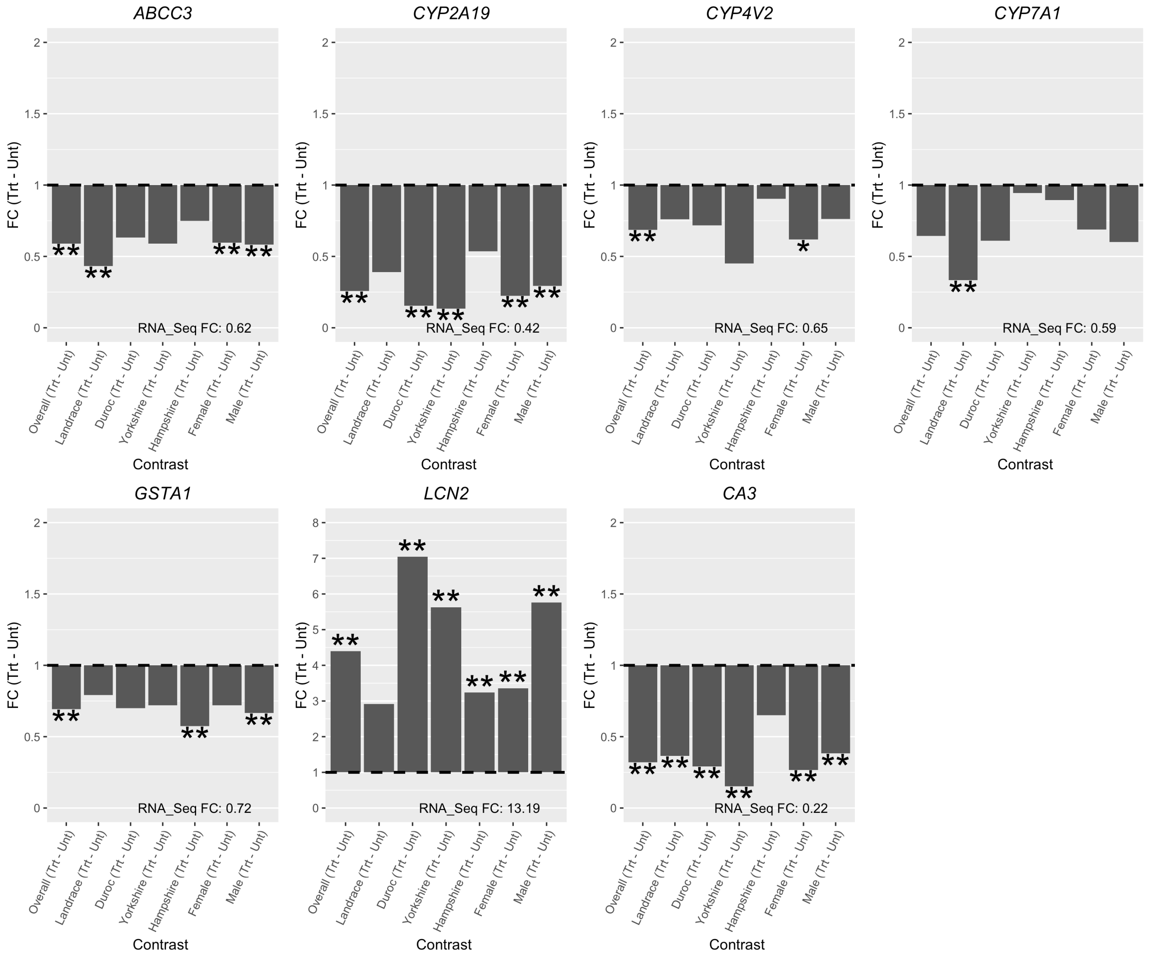

[1] The significance threshold was set at 0.0035 (i.e. **) and a tendency was set at 0.0071 (i.e. *) after the Bonferonni Correction.

[2] Genes up-regulated in treatment have a fold change > 1; Genes down-regulated in treatment fold change < 1.

[3] The RNA Sequence fold change is displayed in the bottom of the figure and the same contrast based on quantitative PCR utilizing the full dataset is the column within each plot (Overall (Trt – Unt)).

Figure S6. Gene dendogram showing the co-expression modules derived from the WGCNA analysis for Fenbendazole and Flunixin meglumine .

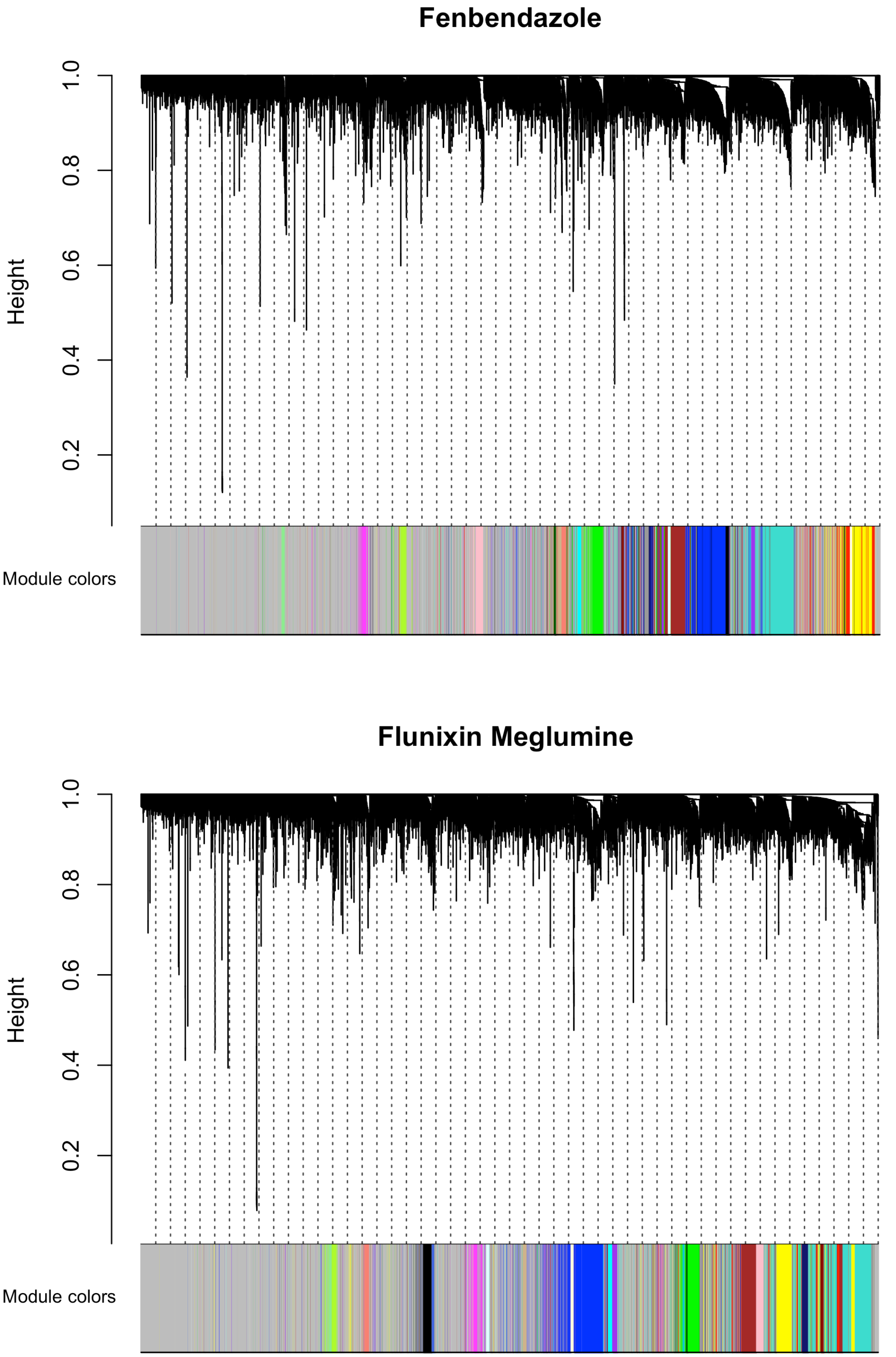

Figure S7. Correlation between modules and pharmacokinetic parameters for Fenbendazole and its metabolite with significant correlations in blue[1].

Module	FBZ_AUC	FBZ_Cl	FBZ_Half_Life	FBZ_MRT	FBZ_VSS	OXF_Cmax	OXF_Tmax
FBZ_yellow	-0.33(0.16)	0.3(0.21)	-0.17(0.48)	-0.33(0.15)	0.22(0.36)	0.04(0.86)	0.32(0.17)
FBZ_turquoise	-0.18(0.45)	0.28(0.23)	-0.08(0.73)	0.01(0.97)	0.42(0.07)	-0.18(0.44)	0.28(0.24)
FBZ_tan	0.16(0.49)	-0.1(0.69)	0.24(0.31)	0.04(0.86)	-0.25(0.3)	0.14(0.56)	-0.44(0.05)
FBZ_salmon	0.21(0.37)	-0.19(0.42)	0.22(0.36)	0.21(0.36)	-0.08(0.75)	0.21(0.38)	-0.49(0.03)
FBZ_royalblue	-0.1(0.68)	0.2(0.39)	-0.18(0.45)	0.1(0.69)	0.29(0.21)	-0.07(0.76)	0.12(0.61)
FBZ_red	-0.17(0.48)	0.1(0.68)	-0.04(0.87)	-0.24(0.32)	0.07(0.75)	0.16(0.5)	0.2(0.39)
FBZ_purple	-0.19(0.42)	0.16(0.5)	-0.13(0.58)	-0.06(0.8)	0.29(0.21)	-0.27(0.25)	0.15(0.52)
FBZ_pink	0.13(0.6)	-0.33(0.15)	0.27(0.25)	-0.05(0.85)	-0.26(0.27)	-0.11(0.63)	-0.18(0.46)
FBZ_midnightblue	0.3(0.2)	-0.34(0.15)	0.14(0.55)	0.18(0.44)	-0.29(0.21)	0.21(0.38)	-0.1(0.66)
FBZ_magenta	-0.11(0.66)	0.22(0.34)	-0.25(0.29)	-0.04(0.85)	0.11(0.65)	0.09(0.7)	0.5(0.03)
FBZ_lightyellow	-0.26(0.27)	0.23(0.34)	-0.16(0.51)	-0.36(0.12)	0.03(0.9)	0.17(0.46)	0.12(0.63)
FBZ_lightgreen	0.05(0.83)	0(0.99)	0.21(0.38)	0.07(0.78)	0.06(0.8)	-0.03(0.91)	-0.02(0.93)
FBZ_lightcyan	-0.12(0.6)	0.23(0.33)	-0.13(0.59)	0.06(0.8)	0.39(0.09)	-0.07(0.78)	0.17(0.46)
FBZ_grey60	0.2(0.4)	-0.25(0.29)	-0.01(0.95)	0.09(0.7)	-0.33(0.15)	0.11(0.63)	0.02(0.95)
FBZ_greenyellow	-0.12(0.61)	0.19(0.42)	-0.25(0.28)	0(0.99)	0.17(0.48)	0.15(0.54)	-0.12(0.63)
FBZ_green	-0.1(0.69)	0.26(0.27)	-0.29(0.21)	0.02(0.93)	0.19(0.43)	0.11(0.63)	0.08(0.72)
FBZ_darkred	-0.3(0.2)	0.22(0.36)	-0.16(0.5)	-0.32(0.17)	0.16(0.5)	-0.09(0.7)	0.2(0.4)
FBZ_darkgreen	0.26(0.26)	-0.31(0.19)	0.27(0.25)	0.23(0.34)	-0.21(0.39)	-0.03(0.9)	-0.56(0.01)
FBZ_cyan	0.3(0.2)	-0.42(0.06)	0.33(0.16)	0.17(0.47)	-0.34(0.14)	-0.08(0.75)	-0.17(0.47)
FBZ_brown	0.32(0.16)	-0.44(0.05)	0.33(0.15)	0.17(0.47)	-0.35(0.12)	0.09(0.71)	-0.28(0.22)
FBZ_blue	0.33(0.16)	-0.34(0.15)	0.17(0.49)	0.19(0.43)	-0.42(0.07)	0.06(0.79)	-0.24(0.31)
FBZ_black	-0.35(0.13)	0.36(0.12)	-0.29(0.21)	-0.25(0.29)	0.31(0.18)	-0.03(0.89)	0.38(0.1)

Phenotype

Non-Signficant Significant

[1] Modules were declared significant if the absolute correlation was greater than 0.30, had a p-value less than 0.10 and at least 10 % of the genes within the module displayed differential transcript levels between the treated versus the controls.

Figure S8. Correlation between modules and pharmacokinetic parameters for Flunixin Meglumine and its metabolite with significant correlations in blue[1].

Module	FLU_AUC	FLU_Cl	FLU_Half_Life	FLU_MRT	FLU_VSS	OH5_CMax
FLU_yellow	0.33(0.16)	-0.42(0.07)	0.28(0.23)	0.13(0.59)	-0.34(0.15)	0.22(0.34)
FLU_turquoise	-0.23(0.32)	0.27(0.25)	-0.22(0.36)	0.05(0.82)	0.3(0.21)	-0.07(0.76)
FLU_tan	-0.15(0.53)	0.12(0.61)	-0.22(0.35)	0.1(0.67)	0.21(0.38)	0.27(0.24)
FLU_salmon	-0.07(0.76)	0.15(0.53)	-0.04(0.87)	0.2(0.41)	0.28(0.23)	-0.17(0.47)
FLU_royalblue	0.2(0.4)	-0.02(0.93)	-0.34(0.14)	-0.6(0.01)	-0.33(0.16)	0.02(0.94)
FLU_red	-0.05(0.83)	0.08(0.73)	0.02(0.95)	0.3(0.2)	0.27(0.25)	-0.3(0.2)
FLU_purple	-0.02(0.95)	0.03(0.9)	0.12(0.63)	0.28(0.24)	0.12(0.61)	-0.34(0.15)
FLU_pink	0.07(0.76)	-0.1(0.68)	0.09(0.71)	0.09(0.71)	-0.04(0.87)	0.15(0.53)
FLU_midnightblue	0.22(0.35)	-0.33(0.16)	0.18(0.46)	0.1(0.68)	-0.25(0.28)	0.37(0.11)
FLU_magenta	-0.08(0.74)	0.05(0.83)	0.11(0.65)	0.2(0.4)	0.18(0.46)	-0.19(0.42)
FLU_lightyellow	0.05(0.82)	-0.2(0.4)	-0.05(0.83)	0.38(0.1)	0.05(0.84)	0.1(0.67)
FLU_lightgreen	-0.06(0.79)	0.11(0.65)	0.11(0.63)	0.08(0.75)	0.05(0.84)	-0.15(0.52)
FLU_lightcyan	0.12(0.61)	-0.1(0.68)	-0.34(0.14)	0.06(0.8)	-0.01(0.97)	-0.03(0.9)
FLU_grey60	-0.04(0.87)	-0.01(0.96)	0.09(0.72)	-0.38(0.1)	-0.23(0.32)	0.21(0.38)
FLU_greenyellow	0.04(0.87)	-0.02(0.94)	0.1(0.66)	0.12(0.62)	-0.04(0.88)	0(0.99)
FLU_green	0.13(0.6)	-0.16(0.51)	0.07(0.76)	-0.36(0.12)	-0.35(0.13)	0.31(0.18)
FLU_darkturquoise	0.23(0.33)	-0.33(0.15)	0.12(0.63)	-0.06(0.82)	-0.34(0.14)	0.47(0.04)
FLU_darkred	-0.21(0.38)	0.23(0.32)	-0.08(0.74)	0.18(0.45)	0.36(0.12)	-0.17(0.47)
FLU_darkgreen	0.13(0.59)	-0.15(0.54)	-0.02(0.92)	-0.28(0.23)	-0.27(0.25)	0.23(0.33)
FLU_cyan	-0.13(0.57)	0.18(0.45)	0.1(0.67)	-0.07(0.76)	0.06(0.82)	-0.21(0.37)
FLU_brown	-0.14(0.55)	0.17(0.46)	-0.04(0.88)	0.04(0.87)	0.16(0.49)	-0.15(0.52)
FLU_blue	0(1)	-0.05(0.84)	-0.01(0.98)	0.13(0.58)	0.05(0.82)	0.35(0.13)
FLU_black	-0.07(0.76)	0.22(0.36)	-0.12(0.61)	-0.6(0.01)	-0.12(0.61)	0.04(0.87)

Phenotype

Non-Signficant | Significant

[1] Modules were declared significant if the absolute correlation was greater than 0.30, had a p-value less than 0.10 and at least 10 % of the genes within the module displayed differential transcript levels between the treated versus the controls.

Figure S9. Heat map of correlations between normalized and batch-adjusted reads per kilobase of transcripts per million-mapped (RPKM) values across genes within the cyan module for Fenbendazole.

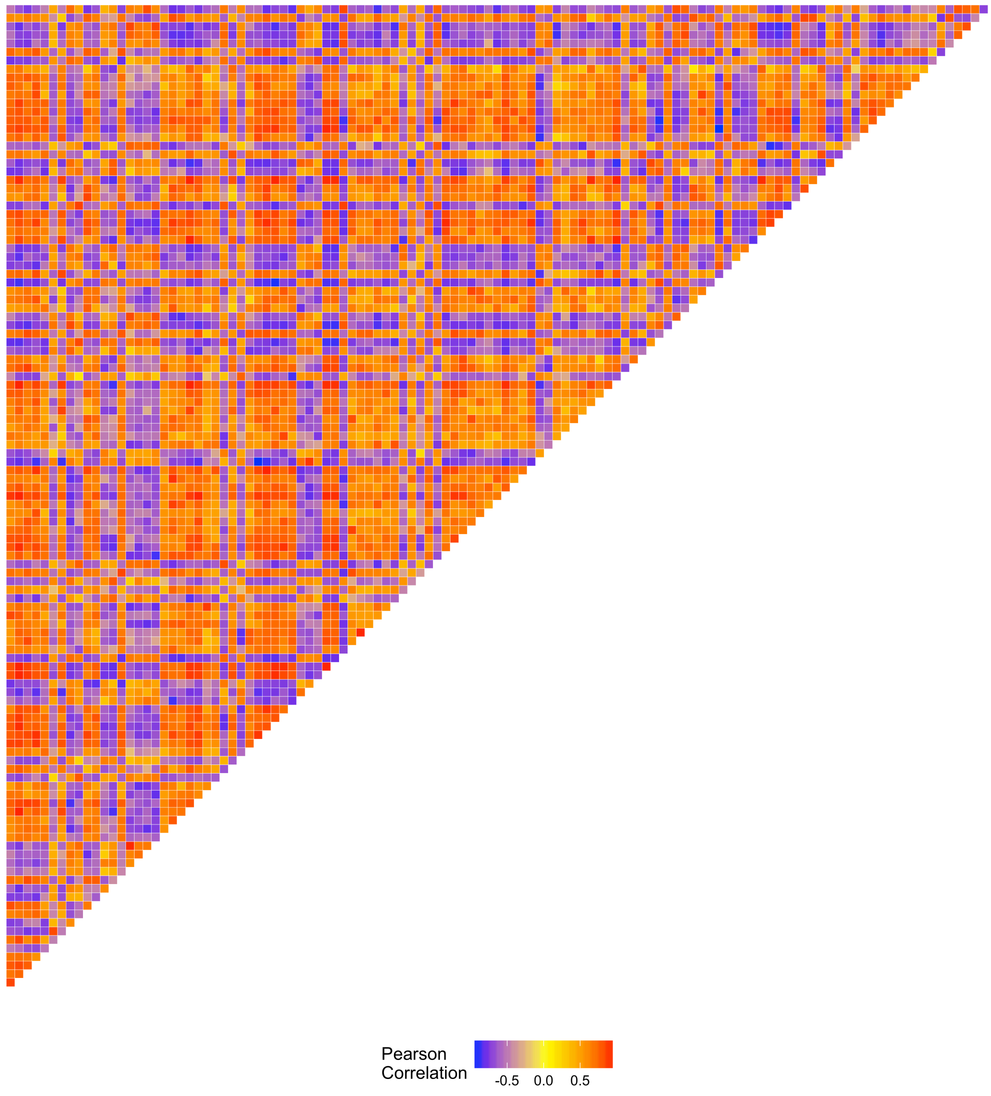

Figure S10. Heat map of correlations between normalized and batch-adjusted reads per kilobase of transcripts per million-mapped (RPKM) values across genes within the brown module for Fenbendazole.

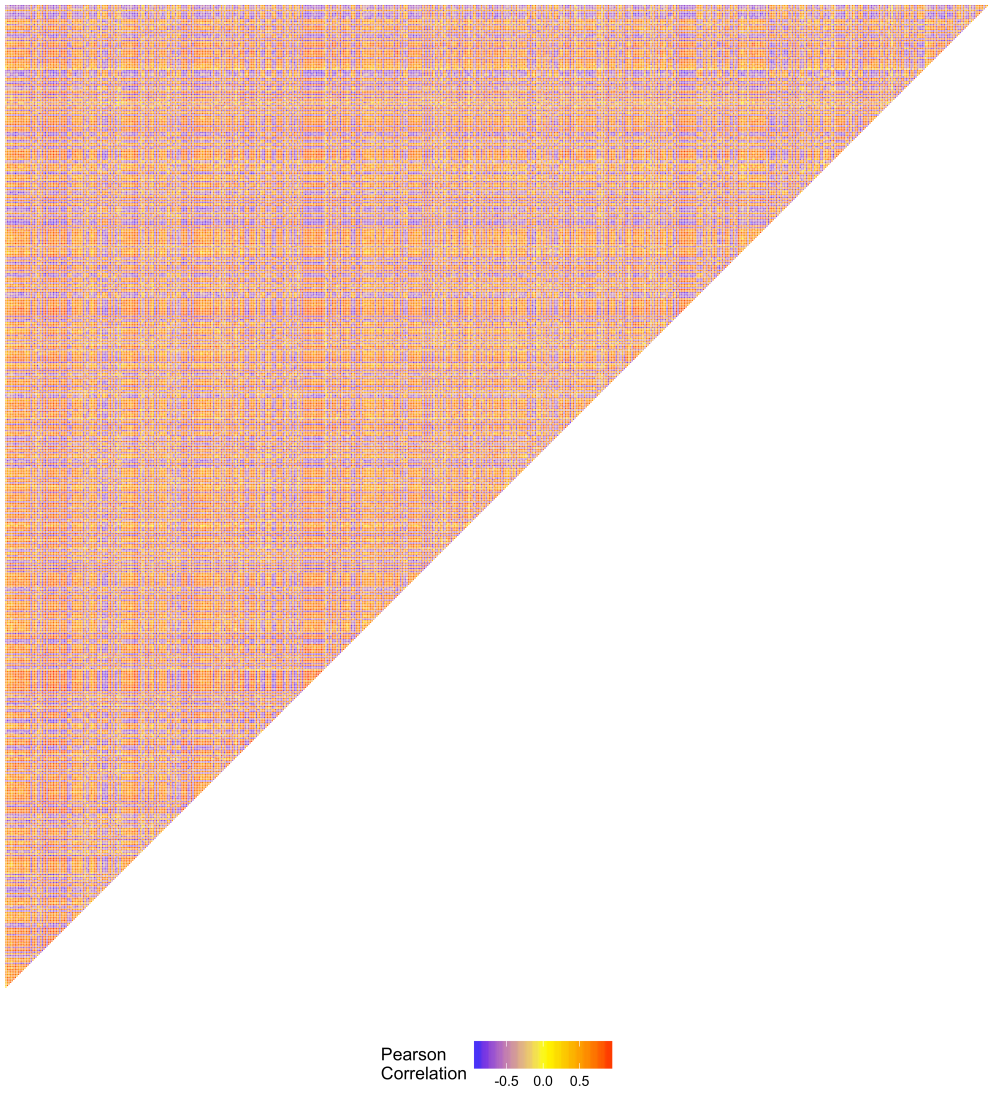

Figure S11. Heat map of correlations between normalized and batch-adjusted reads per kilobase of transcripts per million-mapped (RPKM) values across genes within the magenta module for Fenbendazole.

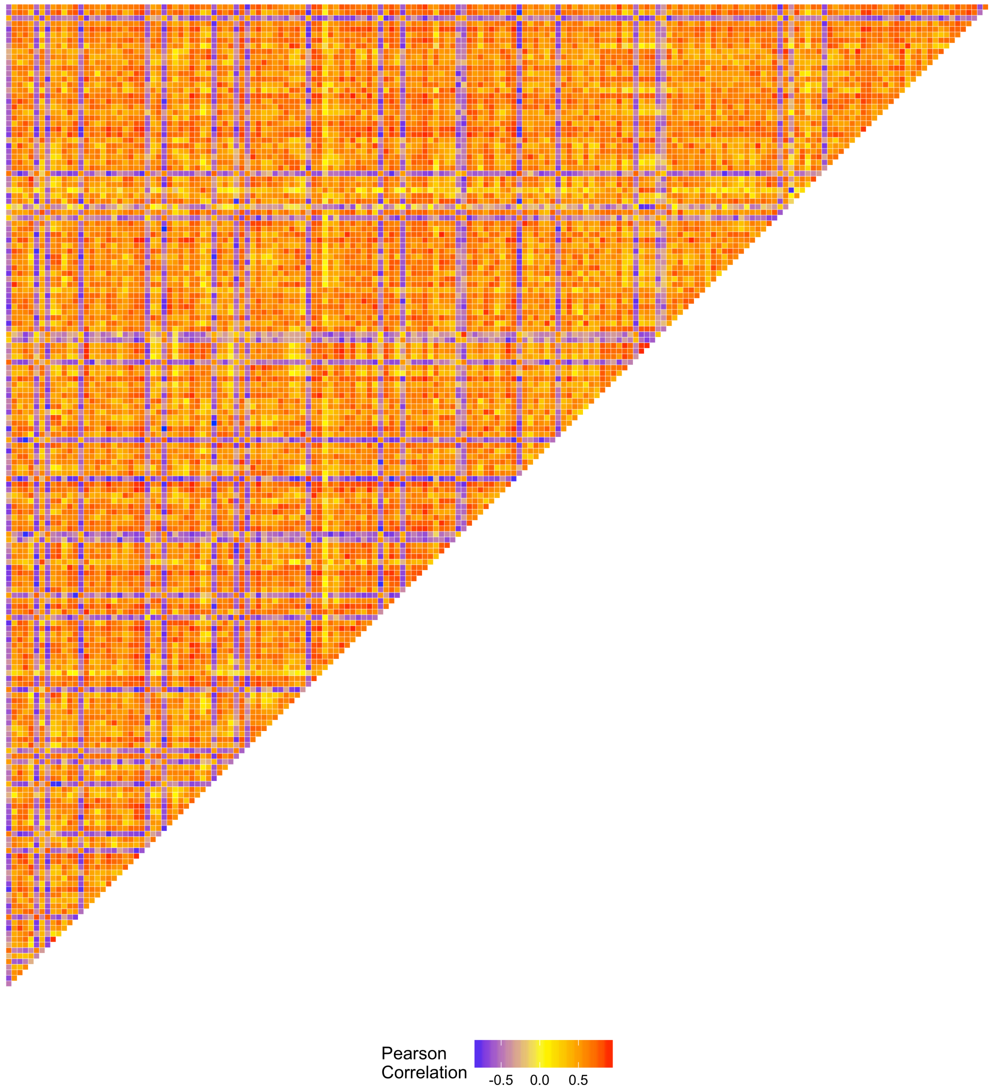

Figure S12. Heat map of correlations between normalized and batch-adjusted reads per kilobase of transcripts per million-mapped (RPKM) values across genes within the grey60 module for Flunixin Meglumine.

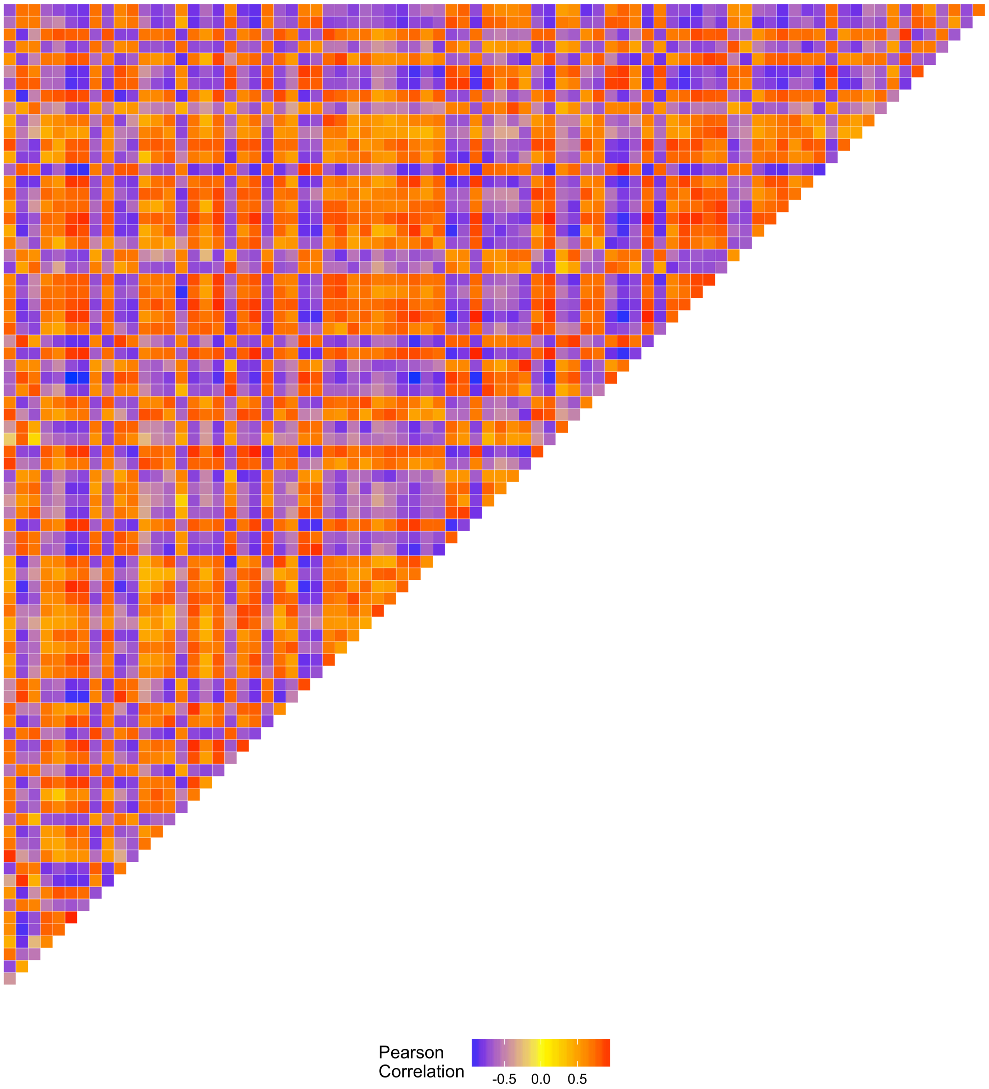

Figure S13. Expression profiles of hub genes across animals administered Flunixin Meglumine (Panel A) and Fenbendazole (Panel B) versus untreated animals within modules associated with pharmacokinetic phenotypes.

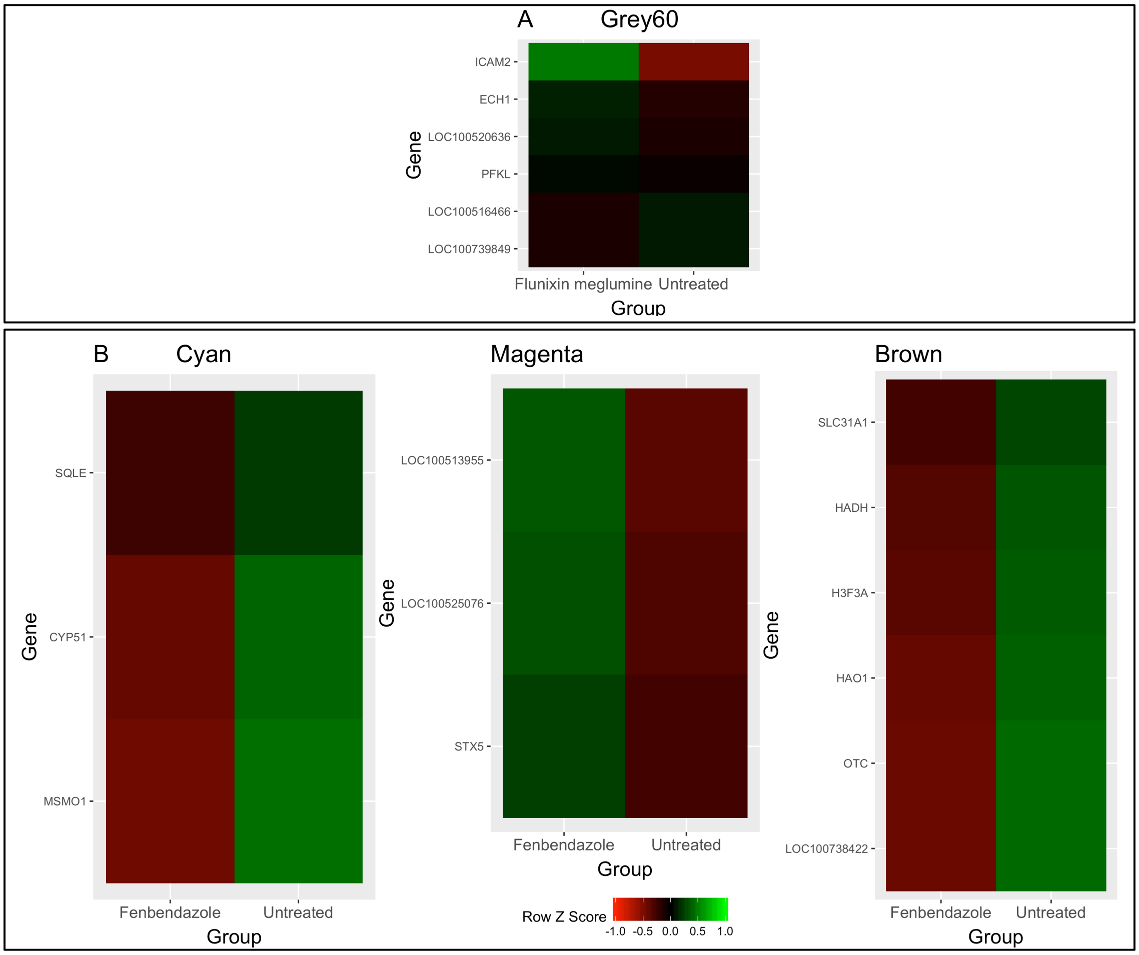

Supplement: Supplementary file 1 — Supplementary Information [file 41598_2017_1526_MOESM1_ESM.pdf]
